# First Metabolomic Signature of Blood-Brain Barrier Opening Induced by Microbubble-Assisted Ultrasound

**DOI:** 10.3389/fnmol.2022.888318

**Published:** 2022-06-20

**Authors:** Antoine Presset, Sylvie Bodard, Antoine Lefèvre, Anaïs Millet, Edward Oujagir, Camille Dupuy, Tarik Iazourène, Ayache Bouakaz, Patrick Emond, Jean-Michel Escoffre, Lydie Nadal-Desbarats

**Affiliations:** ^1^UMR 1253, iBrain, Inserm, Université de Tours, Tours, France; ^2^Département Analyses Chimique et Métabolomique, PST Analyses des Systèmes Biologiques, Université de Tours, Tours, France; ^3^CHRU Tours, Serv Med Nucl in Vitro, Tours, France

**Keywords:** microbubble, blood-brain barrier opening, metabolomics, HPLC-MS, NMR, ultrasound, inflammation, neurotransmission

## Abstract

Microbubble (MB)-assisted ultrasound (US) is a promising physical method to increase non-invasively, transiently, and precisely the permeability of the blood-brain barrier (BBB) to therapeutic molecules. Previous preclinical studies established the innocuity of this procedure using complementary analytical strategies including transcriptomics, histology, brain imaging, and behavioral tests. This cross-sectional study using rats aimed to investigate the metabolic processes following acoustically-mediated BBB opening *in vivo* using multimodal and multimatrices metabolomics approaches. After intravenous injection of MBs, the right striata were exposed to 1-MHz sinusoidal US waves at 0.6 MPa peak negative pressure with a burst length of 10 ms, for 30 s. Then, the striata, cerebrospinal fluid (CSF), blood serum, and urine were collected during sacrifice in three experimental groups at 3 h, 2 days, and 1 week after BBB opening (BBBO) and were compared to a control group where no US was applied. A well-established analytical workflow using nuclear magnetic resonance spectrometry and non-targeted and targeted high-performance liquid chromatography coupled to mass spectrometry were performed on biological tissues and fluids. In our experimental conditions, a reversible BBBO was observed in the striatum without physical damage or a change in rodent weight and behavior. Cerebral, peri-cerebral, and peripheral metabolomes displayed specific and sequential metabolic kinetics. The blood serum metabolome was more impacted in terms of the number of perturbated metabolisms than in the CSF, the striatum, and the urine. In addition, perturbations of arginine and arginine-related metabolisms were detected in all matrices after BBBO, suggesting activation of vasomotor processes and bioenergetic supply. The exploration of the tryptophan metabolism revealed a transient vascular inflammation and a perturbation of serotoninergic neurotransmission in the striatum. For the first time, we characterized the metabolic signature following the acoustically-mediated BBBO within the striatum and its surrounding biological compartments.

## Introduction

The blood-brain barrier (BBB) is one of the most selective and semi-permeable biological barriers in mammals. It isolates the central nervous system (CNS) from the whole body, creating a privileged environment for maintaining the homeostasis of the cerebral compartment (Pardridge et al., 1986). BBB is composed of endothelial cells, perivascular astrocyte end-feet, pericytes, neurons, and basal membrane. The permeability of this barrier is drastically reduced by the presence of tight junctions (TJs) between endothelial cells, abolishing any paracellular diffusion of molecules (Abbott et al., 2010). In addition, brain cells and blood vessel cells are provided with passive and active transporters that regulate the concentration of molecules within the cerebral tissue. Nutrients, gases (O_2_ and CO_2_), ions, water (through aquaporin-4), and small lipid-soluble molecules less than 400 Daltons can pass through this barrier by facilitated diffusions, such as glucose and large amino-acid transporters (Daneman and Prat, 2015). Nevertheless, active efflux proteins (ATP-binding cassette and solute carrier family transporters) transport any compound from the brain tissue to the vascular compartment. These active transporters limit the passage through the BBB of pathogens, toxins, solutes, and large hydrophilic molecules like therapeutics (Barar et al., 2016). Because of this barrier and its physiological functions, the BBB renders the treatment of neurological diseases challenging by reducing, if not abolishing, the biodistribution of therapeutics in the cerebral tissue. One way to bypass this barrier is to transiently disrupt endothelial cells by applying the microbubble (MB)-assisted ultrasound (US) method (also known as sonoporation), thus allowing a non-invasive and targeted delivery of therapeutics. MBs are intravenously injected and locally activated by focused US, thus increasing the permeability of the vascular endothelium and facilitating drug extravasation and its bioavailability in the targeted brain tissue (Presset et al., 2020). Importantly, this procedure enables precise BBB opening (BBBO) in deeper brain regions without resorting to surgical intervention. For instance, the striatum is a functional deep subcortical nucleus in the brain involved in the processing of motor and cognitive tasks. This cerebral structure is specifically implicated in several neurological and psychiatric pathologies, such as Parkinson’s or Huntington’s disease, addiction, bipolar or autism spectrum disorders, and attention deficit or hyperactivity disorders (Grahn et al., 2008). Hence, the striatum remains a prime target for studying the effect of neuroprotective treatment (Schober, 2004) or novel curative strategies, such as cellular therapies (Gaillard and Jaber, 2011).

Blood-brain barrier opening safety using US and MBs has been investigated using complementary analytical strategies including transcriptomics, histology, behavioral tests, and brain imaging, e.g., magnetic resonance imaging (MRI) and positron emission tomography/computed tomography (PET/CT). Transcriptomic studies showed overexpression of pro-inflammatory- and antiapoptotic-associated genes (Kovacs et al., 2017; McMahon and Hynynen, 2017; McMahon et al., 2017, 2020; Ji et al., 2021). Gene set enrichment analysis allowed describing classes of genes that are over- or under-represented in a large set of genes. These results revealed the involvement of drug transporter activity, chronic inflammatory response, and antigenic stimuli associated with the inflammatory response. Immunohistochemistry (IHC) analysis of the brain tissue partly confirmed these results. Indeed, this approach revealed the presence of inflammatory proteins and scavenger receptors of complex hemoglobin/haptoglobin, an over-expression of glial fibrillary acidic protein (GFAP), and some apoptotic neurons. Electron microscopy allowed a deep investigation of TJ integrity and the cytoarchitectural consequences of acoustically-mediated BBBO, especially on clathrin- and caveolin-mediated endocytosis and paracellular adhesion. In addition, hematoxylin and eosin staining of brain sections only showed rare red-blood-cell extravasation and gliosis under severe acoustic conditions (Sheikov et al., 2004, 2006, 2008; McDannold et al., 2005; Baseri et al., 2010; Alonso et al., 2011; Kovacs et al., 2018), namely a very high dose of MBs (corresponding to 10-fold the conventional MB dose applied in clinical US imaging, i.e., 100 μL of Definity MBs/kg) or a high mechanical index of 100. Several imaging studies, such as brain MRI, have confirmed the absence of hemorrhages or edema during the BBBO procedure under clinical acoustic conditions (Liu et al., 2008; Kim et al., 2013; Horodyckid et al., 2017; Mainprize et al., 2019). Behavioral studies on non-human primates (NHP) or rodents did not show significant differences between the experimental groups (Olumolade et al., 2016; Tsai et al., 2018). All these studies convey that acoustically-mediated BBBO induces low or no neurophysiological damage onto the targeted tissue. These potential damages are directly correlated to the acoustic parameters and MBs, i.e., their composition and concentration, specifically (McMahon and Hynynen, 2017; McMahon et al., 2019). To date, to the best of our knowledge, no study explored the neurophysiological aftermath of this procedure on the metabolic level. In addition, all studies cited above focused on the cerebral tissue or endothelial cells. The vascular compartment has been shown to be the first impacted matrix after BBBO by MB-assisted US (Presset et al., 2020). Therefore, the present study focused on the metabolomic fingerprint of the brain tissue and body fluids, such as the cerebrospinal fluid, the blood, and urine following acoustically BBBO.

Metabolomics is an omics-based discipline that investigates, at a large scale, small molecules (called metabolites) from metabolic processes of cells, biofluids, tissues, and organisms defined as biological matrices. This promising and powerful approach gives access to the molecular phenotype by identifying the metabolites and measuring their concentrations, thus enabling the direct readout of the underlying biochemical activity in contrast to other omics approaches (e.g., genomics, transcriptomics, among others). These metabolites and their interactions within a given biological system are defined as the metabolome. To cover most of the metabolome, a multiplatform metabolomics approach, including nuclear magnetic resonance (NMR) spectroscopy and mass spectroscopy (MS), is often implemented and allows exploring the influence of physical factors on the functioning of an organism by the description of its metabolome. Metabolomics showed a clear potential in the evaluation of biomarkers for diagnosis, prognosis, and therapeutic monitoring. In this context, the present study aimed to investigate the metabolic consequences of acoustically-mediated BBBO targeted in the striatum region. As already described in transcriptomics approaches (Kovacs et al., 2017, 2018; McMahon and Hynynen, 2017; McMahon et al., 2017, 2020), short longitudinal studies were conducted; specifically, the maximum interval reported between sonoporation and transcriptomic investigations was 4 days after acoustically-mediated BBBO (McMahon and Hynynen, 2017). In this present study, a wide longitudinal study (up to a week after the procedure) of these metabolic consequences on brain tissue (i.e., striatum), cerebrospinal fluid (CSF), blood serum, and urine metabolomes was performed using NMR spectroscopy and high-performance liquid chromatography coupled with mass spectrometry (HPLC-MS).

## Materials and Methods

### Animals and Housing

All male rats Sprague Dawley (Janvier Labs, Le Genest-Saint-Isle, France) were 7 weeks old (about 250 g) before experiments. They were housed in groups of 4 under humidity and temperature-controlled conditions and a 12:12 light-dark cycle (light on at 7:00 AM) with *ad libitum* access to food and water. Animals were acclimated to their housing for 1 week before the *in vivo* procedures.

### Acoustically-Mediated Blood-Brain Barrier Opening

#### Ultrasound Set-Up

As previously described, US waves were generated using a single element lab-made transducer with a center frequency of 1 MHz (Escoffre et al., 2013). The transducer had a diameter of 49 mm and was focused at 49 mm. It was driven with an electrical signal generated by an arbitrary waveform generator (Ref. 33220A; Agilent Technologies Inc., Santa Clara, CA, United States), then amplified with a power amplifier (Ref. AAP-500-0.2-6-D; ADECE, Vallauris, France). The peak negative pressure was determined in a distinct setup using a capsule hydrophone (Ref. HGL-0200; ONDA Corporation, Sunnyvale, CA, United States) at the focal distance of the transducer.

#### Blood-Brain Barrier Opening Protocol

**FIGURE 1:**
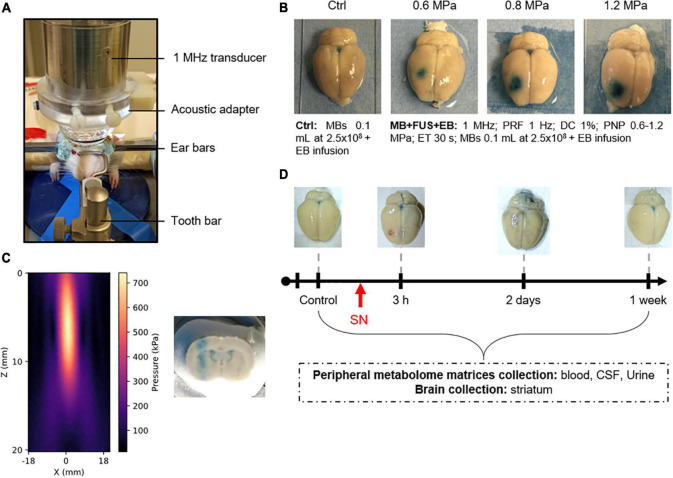
Effects of acoustic pressure on BBB opening (BBBO) without massive hemorrhages and damages. **(A)** Ultrasound (US) device. **(B)** Determination of optimal acoustic pressure for BBBO. **(C)** Ultrasonic beam profile in lateral and depth dimensions of the focal spot in free-gas water. A coronal slice was also cut after Evans Blue dye infusion to visualize the focal spot. **(D)** Timeline of animal experiments. SN means “sonoporation.”

Rats were anesthetized with a mixture of ketamine (70 mg/kg, Kétamine 1000^®^, Virbac, Carros, France) and xylazine (7 mg/kg, Rompun^®^, Elanco, Cuxhaven, Germany). Then, they were placed into a stereotactic frame and on a heating plate. The 1 MHz single element transducer (Figure 1A) was inserted in a degassed water-filled cone to ensure coupling with the head of the animals. This transducer was placed over the scalped skull at specific stereotactic coordinates of the region of interest (ROI), the right striatum, i.e., anteroposterior – AP −0.5 mm, Lateral – L ± 3.15 mm, and ventrodorsal – VD 5 mm. From now on, the right striatum will be called striatum ipsilateral. After a bolus injection of MBs (Vevo Micromarker; 100 μL at 2.5 × 10^8^ MBs/mL; mean diameter in volume 2.3 to 2.9 μm; Fujifilm-Visualsonics Inc., Amsterdam, Netherlands), the ROI was exposed to 1 MHz sinusoidal US waves for 30 s with a pulse repetition frequency (PRF) of 1 Hz and 10,000 cycles per pulse (10 ms burst length). The peak negative pressure (PNP) ranged from 0 to 1.2 MPa in agreement with Gerstenmayer et al. (2018). Immediately after US exposure, a 2% Evans blue solution (EB; Sigma-Aldrich, St. Louis, MO, United States) was carefully intravenously injected at 5 ml/kg *via* the caudal vein to monitor the BBBO. After 1 h, animals were intraperitoneally injected with an overdose of the anesthetic mixture and transcardially perfused with a 0.9% saline solution. Then, brains were collected and observed under an optical stereomicroscope (MZ9.5; Leica, Wetzlar, Germany).

For metabolomics analysis, BBBO was achieved one time using the same protocol above described using a PNP of 0.6 MPa but the EB solution was not administered. As defined above, the animals were sacrificed for 3 h (3-h group), 2 days (2-days group), and 1 week (1-week group) after the BBBO. Animals from control groups were treated as described above with no US application. The group size is presented in Table 1.

**TABLE 1:** Summary of sample sizes by matrix and group and of animal weight (before and after the sonoporation).

		Number of samples			
	Group	Control	3-h	2 days	1 week
Matrices	Striatum	8	8	8	8
	CSF	8	8	7	8
	Blood serum	11	10	9	10
	Urine	8	8	8	10
Weight (g)	Before MB-FUS	343 ± 15	332 ± 11	341 ± 22	344 ± 24
	After MB-FUS	367 ± 19	383 ± 22	378 ± 28	379 ± 28

*Bodyweight of rats is expressed as mean ± standard deviation. MB-FUS: microbubble-assisted focused ultrasound.*

Animal wellbeing has been assessed each day until sacrifice by monitoring variations in rat behaviors (i.e., respiratory deficiency, loss of locomotion, loss of appetite, loss of grooming, aggressiveness, fear, etc.) and in body weights. Rat weights before and after the procedure have been consigned in Table 1.

### Collection and Preparation of Biological Samples

#### Striatum

Brains tissues were collected and striata were dissected by an expert in brain microsurgery. Then, tissues were lyophilized and homogenized. Dry residues were weighted to approximately 3 mg. Finally, metabolites were extracted using an extraction protocol described by Dieme et al. (2017).

#### Cerebrospinal Fluid

Cerebrospinal fluid (CSF) was collected from cisterna magna and was centrifugated at 10,000 *g* for 15 min at 4°C. Finally, the extraction of metabolites was performed according to a protocol established by Blasco et al. (2013).

#### Blood Serum

Blood was collected directly from the right cardiac atria and was coagulated for 30 min at room temperature. Then, blood was centrifugated at 10,000 *g* for 15 min at 4°C. The supernatant was collected corresponding to blood serum. Metabolites were extracted as previously reported by Alshammari et al. (2015).

#### Urine

Urine was collected directly from the output of the bladder. For ^1^H NMR analyses, samples were centrifuged and diluted to ^1^/_4_ in D_2_O with final concentration of 123 μM of trimethylsilylpropanoic acid (TSP) buffer (Dieme et al., 2015).

### 1H Nuclear Magnetic Resonance Spectroscopy and High-Performance Liquid Chromatography Coupled With Mass Spectrometry Analysis

As previously reported by Dieme et al. (2015, 2017) ^1^H NMR analyses were carried out on brain tissue, CSF, blood serum, and urine. Briefly, these analyses were performed on a Bruker DRX-600 Avance III HD (Bruker, Billerica, MA, United States) equipped with a TCI cryoprobe. Spectra were acquired using a “noesypr1d” pulse sequence with a relaxation delay of 20 s. Scans were acquired successively 64 times on each sample.

Striata, CSF, and blood serum were analyzed using HPLC-MS analysis as established by Dupuy et al. (2021). In brief, HPLC-MS analyses were performed on a UHPLC Ultimate WPS-3000 system (Dionex, Sunnyvale, CA, United States) coupled to a Q-Exactive mass spectrometer (Thermo Fisher Scientific, Bremen, Germany) and operated in positive (ESI+) and negative (ESI-) electrospray ionization modes. HPLC was achieved using two different columns to increase the metabolic coverage: a Kinetex XB C18 column (150 mm × 2.1 mm × 1.7 μm; Phenomenex Inc., Torrance, CA, United States) and a Cortecs HILIC column (150 mm × 2.1 mm × 1.6 μm; Waters Corporation, Milford, MA, United States). During the full-scan acquisition (full MS, AGC target of 10^6^, maximum injection time of 250 ms), which ranged from 58 to 870 m/z, the instrument operated at 70,000 resolution (m/z = 200). Finally, tryptophan’s derivates were quantified within brain tissues, CSF, and blood serum as described in Lefevre et al. (2019); Alarcan et al. (2021).

### Data Processing

#### Metabolite Identification

A targeted analysis was applied to the samples of each matrix, based on a library of standard compounds (Mass Spectroscopy Metabolite Library of standards; IROA Technologies, Sea Girt, NJ, United States). The following criteria were used to identify the metabolites: retention time of the detected metabolite within ± 20 s of the standard reference and exact measured molecular mass of the metabolite within a range of 10 ppm around the known molecular mass of the reference compound. The intensity value was calculated using Xcalibur 2.2 software (ThermoFisher Scientific, San Jose, CA, United States) by integrating the chromatographic peak area corresponding to the selected metabolite. In addition, tryptophan derivates were identified and quantified by the correspondence between isotopic ratios of the metabolite and the labeled internal standard. NMR spectra were preprocessed (phase correction, baseline correction, peak alignment, and normalization), and NMR peak identification and quantification were performed by fitting spectra with a library of pure quantified metabolite spectrum with R software 4.0.2 (R Foundation for Statistical Computing, Vienna, Austria) with ASICS package 2.6.1 (Lefort et al., 2019).

#### Normalization

Concentration and intensities of blood serum and CSF samples were normalized to the total area while cerebral tissue samples were normalized to the weighted dry masses. Creatinine concentration was determined using TSP as an internal standard compound. Urine samples were normalized to the creatinine concentration (Craig et al., 2006).

#### Quality Control

High-performance liquid chromatography coupled with mass spectrometry and ^1^H NMR instruments’ stability were evaluated by multiple injections of quality control (QC) samples obtained from a pool of 10 μl of all samples analyzed. QCs were analyzed at the beginning of the run, every 10 samples, and at the end of the run. Coefficients of variation, defined as CV = (the standard deviation/mean) × 100, were calculated for all metabolites in the dataset. Metabolites detected with a CV greater than 30% in QC samples were discarded for further analysis.

#### High-Performance Liquid Chromatography Coupled With Mass Spectrometry and 1H Nuclear Magnetic Resonance Spectroscopy Data Fusion

Each biological matrix was explored using HPLC-MS and ^1^H NMR. For CSF samples, 181 metabolites in C18 column ESI+/ESI-; 119 metabolites in HILIC column ESI+, and 86 metabolites in NMR spectroscopy, were identified. For blood serum samples, 306 metabolites in C18 column ESI+/ESI-; 160 metabolites in HILIC column ESI+, and 109 metabolites in NMR spectroscopy, were identified. For brain samples, 224 metabolites in C18 column ESI+/ESI-; 161 metabolites in HILIC column ESI+, and 92 metabolites in NMR spectroscopy, were identified. Each metabolite of lists coming from the different platforms and modalities was associated with universal identifiers from online databases, such as Kyoto Encyclopedia of Genes and Genomes (KEGG, Kyoto, Japan, accessed on 10 June 2021), Human Metabolome Database (HMDB, Ottawa, Canada, accessed on 10 June 2021), and PubChem (Bethesda, MD, United States, accessed on 10 June 2021) databases (Kanehisa et al., 2017; Wishart et al., 2018; Kim et al., 2021). The redundancy of unique identifiers was analyzed to keep only metabolites with the smallest CV found in the 4 explored modalities (C18-MS ESI+/ESI-, HILIC-MS ESI+, and NMR spectroscopy). After redundancy filtering, 214 metabolites were identified in CSF, 251 were identified in blood serum, and 199 were identified in the striatum. The number of identified metabolites only with NMR spectroscopy in urine was 124.

#### Statistical Analyses

Multivariate data analyses were achieved using unsupervised principal component analysis (PCA). PCA is commonly used for dimensionality reduction by projecting each data point onto only the first few principal components to obtain lower-dimensional data while preserving as much of the data’s variation as possible. It allows summarizing data by reducing the number of variables and denoising the data. In addition, this statistical approach has several goals: (i) exploration of a large dataset characterized by several quantitative variables; (ii) identification of clusters by grouping samples that share nearly metabolic profiles, represented by score plot; and (iii) identification of aberrant samples called outliers. Supervised partial least square discriminant analysis (PLS-DA) was also used to establish predictive models between two-time groups by constraining the sample membership to specific groups. The model’s significance is evaluated by two *p*-values: pR2Y that assesses the significance of the model and pQ2 that reflects the predictability of the model. These supervised analyses determine the metric termed variable importance in projection (VIP), which summarize the importance of each metabolite to define the predictive model. All of these multivariate analyses were performed using ropls R package 1.22 (Thevenot et al., 2015). After checking the normal distribution of samples (Shapiro test) and the homogeneity of variance (Fligner test), non-parametric pairwise multiple comparisons in independent groups using Dunn’s test with Bonferroni *p*-value adjustment were performed. Metabolites were considered significant when the adjusted *p*-value was inferior to 0.05. Finally, significant metabolites identified by univariate analyses depending on the temporality comparisons were used to perform metabolic pathway analyses using a hypergeometric test with false discovery rate (FDR) *p*-value adjustment, based on definitions of the KEGG database. Each significant pathway (i.e., adjusted *p*-value inferior to 0.05) was reviewed to verify its pertinent involvement in the explanation of biological differences (i.e., metabolites that follow pertinent metabolic ways and biochemical reactions with other dysregulated metabolites).

## Results

### Reversible Blood-Brain Barrier Opening Without Inducing Permanent Tissue Damages

Particular attention has been paid to animal well-being. The procedure is well tolerated by animals that did not show apparent physical signs of suffering and have not lost weight (Table 1) before and after the US application. The BBBO was assessed by EB extravasation immediately after the intravascular injection (i.v.) of MBs with or without US exposure. The results are shown in Figure 1B. As expected, the i.v. injection of MBs alone (i.e., control condition) induced neither an EB extravasation nor microhemorrhages in the target area. However, the exposure of this target area to the MB-assisted US at 0.6 MPa resulted in EB extravasation, thus confirming the BBBO. The qualitative microscopic analysis of the brain tissue revealed the occasional presence of a few microhemorrhages. The increase of acoustic pressure from 0.8 to 1.2 MPa induced an enhancement of the tissue area where EB was extravasated. This BBBO was associated with a moderate to a high number of microhemorrhages at both 0.8 and 1.2 MPa, respectively. Taking this into consideration, an acoustic pressure of 0.6 MPa was retained for the investigation of metabolic consequences of acoustically-mediated BBBO using metabolomics approaches.

To assess the duration of BBBO at 0.6 MPa, EB was intravenously injected at different time points (immediately to 1 week) after the US exposure and then brains were collected and analyzed. Our data showed that the native BBB permeability was restored 1-h post-US exposure (Supplementary Figure 1). Few microhemorrhages were observed until 3 h after BBBO only (Figure 1D). As shown in Figure 1, the results demonstrate that our protocol induced a reversible BBBO without inducing irreversible damage to the tissue.

### Multiplatform and Multimatrices Cross-Analyses Allow the Identification of Common Metabolites Between Matrices

**FIGURE 2:**
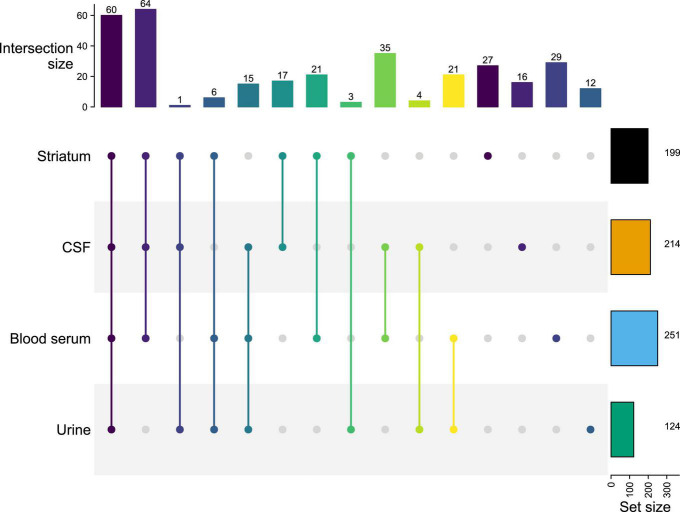
Upset diagram based on metabolites identified within explored matrices (striatum, CSF, blood serum, and urine). Intersection size corresponds to the number of common metabolites detected in different matrices (e.g., on the second column striatum, CSF, and blood serum matrices share 64 metabolites). Set size corresponds to the total number of metabolites per matrix.

As previously reported, an upset diagram of identified metabolites depending on the degree mode of intersections was generated to assess the complementarity in terms of metabolite coverage across all matrices (Figure 2; Lex et al., 2014). This diagram allows efficient identification of the total number of metabolites per matrix (i.e., set size) and the number of common metabolites detected in different matrices (i.e., intersection set). In Figure 2, the horizontal bar graph indicates the total number of metabolites for each matrix: 124 in urines, 199 in the striatum, 214 in the CSF, and 251 in the blood serum. As expected, the blood serum metabolome provides the highest specificity. Indeed, the blood serum, and by extension the blood, is a biological matrix well known to be rich in metabolites (Psychogios et al., 2011).

Supplementary Table 5 shows the detailed list of metabolites identified in each intersection of the upset diagram. As shown in Figure 2, the vertical bar graph indicates the total number of only exclusive metabolites with one biological matrix: 12 in urines, 16 in the CSF, 27 in the striatum, and 29 in the blood serum. The presence of these unique metabolites displays the complementarity of the metabolic coverage supplied by each biological matrix. With 35 common metabolites, CSF and blood serum provide a higher score of intersection between the two matrices. This result is not surprising given the expected correlation between the CSF and the blood serum. Indeed, the CSF is an ultrafiltrate of blood, which is substantially similar to it, except that CSF is a nearly protein-free matrix, which has some different electrolytes concentrations (Segal, 2000; Bulat and Klarica, 2011). As expected, the intersection with brain tissue, CSF, and blood serum provides a higher number of metabolites shared between the three matrices (i.e., 64 metabolites). Indeed, these compartments are directly related to each other through biological barriers (i.e., blood-CSF barrier, glomerular filtration, etc.).

### Cerebral and Peripheral Metabolomes Depict Specific and Sequential Kinetics After Acoustically-Mediated Blood-Brain Barrier Opening

**FIGURE 3:**
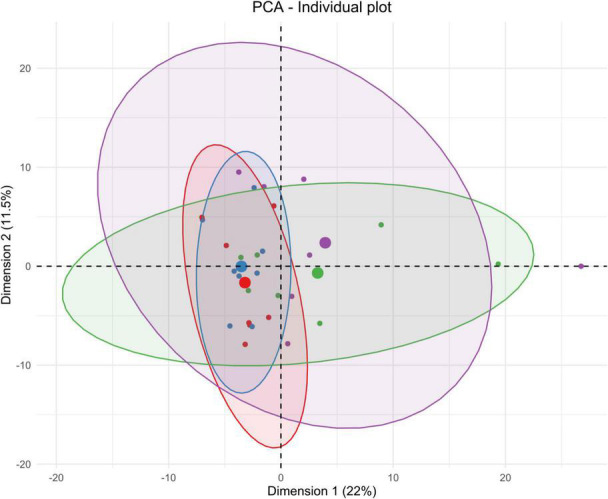
Unsupervised multivariate analyses on striatum metabolome between control and insonified groups. Score plot of principal component analysis (PCA) constructed with metabolites within the striatum metabolome after acoustically-mediated BBB opening (BBBO). Larger dots are inertial centers of ellipses (center of a sample group) plotted based on smaller dots representing each sample individually. Red dots are control samples; Blue dots are 3-h post-BBBO samples; Green dots are 2-days post-BBBO samples; Purple dots are 1-week post-BBBO samples. Two samples having the same metabolic profile will be clustered in the same dimensional space.

**FIGURE 4:**
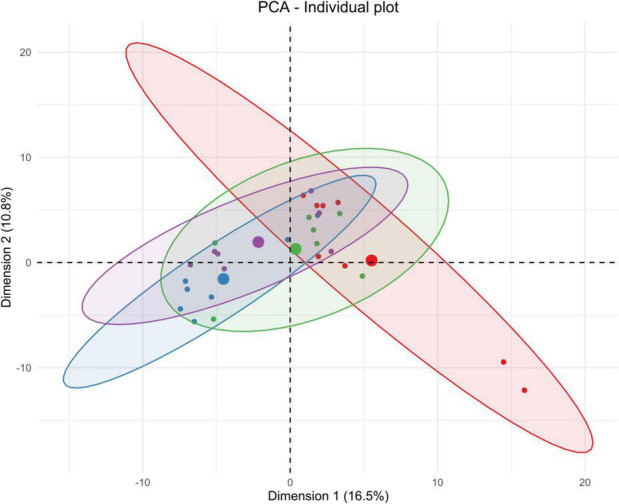
Unsupervised multivariate analyses on CSF metabolome between control and insonified groups. Score plot of principal component analysis (PCA) constructed with metabolites within the CSF metabolome after acoustically-mediated BBB opening (BBBO). Larger dots are inertial centers of ellipses (center of a sample group) plotted based on smaller dots representing each sample individually. Red dots are control samples; Blue dots are 3-h post-BBBO samples; Green dots are 2-days post-BBBO samples; Purple dots are 1-week post-BBBO samples. Two samples having the same metabolic profile will be clustered in the same dimensional space.

**FIGURE 5:**
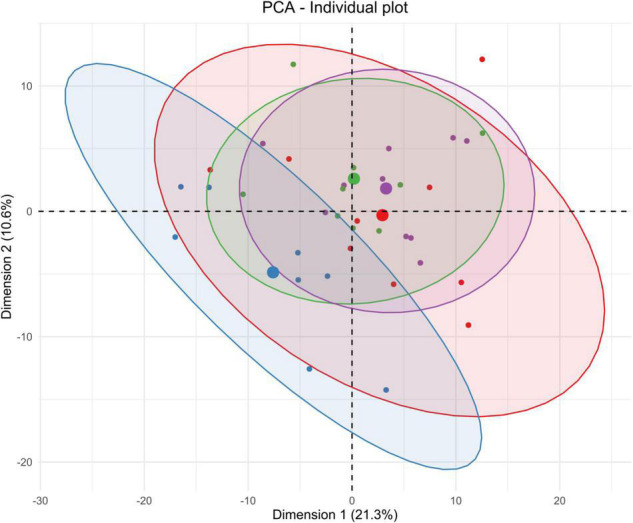
Unsupervised multivariate analyses on blood serum metabolome between control and insonified groups. Score plot of principal component analysis (PCA) constructed with metabolites within the blood serum metabolome after acoustically-mediated BBB opening (BBBO). Larger dots are inertial centers of ellipses (center of a sample group) plotted based on smaller dots representing each sample individually. Red dots are control samples; Blue dots are 3-h post-BBBO samples; Green dots are 2-days post-BBBO samples; Purple dots are 1-week post-BBBO samples. Two samples having the same metabolic profile will be clustered in the same dimensional space.

**FIGURE 6:**
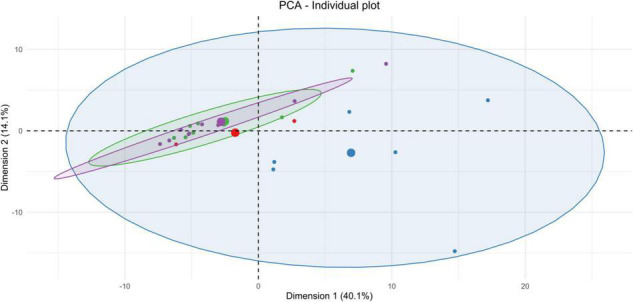
Unsupervised multivariate analyses on urine metabolome between control and insonified groups. Score plot of principal component analysis (PCA) constructed with metabolites within the urine metabolome after acoustically-mediated BBB opening (BBBO). Larger dots are inertial centers of ellipses (center of a sample group) plotted based on smaller dots representing each sample individually. Red dots are control samples; Blue dots are 3-h post-BBBO samples; Green dots are 2-days post-BBBO samples; Purple dots are 1-week post-BBBO samples. Two samples having the same metabolic profile will be clustered in the same dimensional space.

The metabolomes’ profiles of different biological matrices (e.g., striatum, CSF, blood serum, and urine) from control and sonicated groups at different time points (e.g., 3-h, 2-days, 1-week) post-BBBO were determined by using unsupervised multivariate analyses (PCA). Taking into consideration the temporal dimension of these profiles, the kinetics of metabolic changes after acoustically-mediated BBBO for each matrix is defined. As shown in Figures 3–6 and Tables 2–5, each matrix showed specific kinetics of metabolic changes. The blood serum metabolome was more important than CSF, striatum, and urine metabolomes in terms of observed metabolic perturbations. Indeed, the CSF metabolome seems to return to the basal level more quickly than the blood serum metabolome. There is no longer any metabolic disruption in CSF 3 h after BBBO in comparison with the blood serum metabolome, which showed metabolic perturbations up to 1-week post-BBBO. In contrast, metabolic dysregulations within the striatum metabolome were greatest 2 days post-BBBO and persisted for up to a week, as well as urine metabolome showed metabolic perturbations up to a week after the BBBO procedure.

**TABLE 2:** Supervised multivariate PLS-DA models computed from metabolites found in striatum metabolome differentiating two groups.

Model	R2X	R2Y	Q2	pR2Y	pQ2	VIP >1
Control vs. 3-h	0.410	0.988	0.767	0.15	0.05	90
3-h vs. 2-days	0.396	0.991	0.764	0.10	0.05	113
2-days vs. 1-week	0.423	0.991	0.389	0.15	0.35	84
1-week vs. Control	0.491	0.981	0.782	0.40	0.05	100
Control vs. 2-days	0.416	0.995	0.763	0.10	0.20	98
3-h vs. 1-week	0.448	0.981	0.643	0.10	0.10	100
						

*Model is considered significant if permutation metrics (pR2Y and pQ2, corresponding to the p-value of the permutation test) are inferior to 0.05 (in bold), as well as predictive metric (Q2) is higher than 0.5. Variable importance in projection (VIP) is exploitable if and only if the predictive model is significant. Only VIP superior to 1 has been counted (VIP > 1 column).*

**TABLE 3:** Supervised multivariate PLS-DA models computed from metabolites found in CSF metabolome differentiating two groups.

Model	R2X	R2Y	Q2	pR2Y	pQ2	VIP >1
Control vs. 3-h	0.504	0.992	0.848	**0.05**	**0.05**	86
3-h vs. 2-days	0.426	0.993	0.828	**0.05**	**0.05**	80
2-days vs. 1-week	0.407	0.959	0.171	0.80	0.60	74
1-week vs. Control	0.516	0.959	0.426	0.45	0.15	94
Control vs. 2-days	0.445	0.976	0.352	0.10	0.20	93
3-h vs. 1-week	0.397	0.998	0.840	0.15	0.05	71

*Model is considered significant if permutation metrics (pR2Y and pQ2, corresponding to the p-value of the permutation test) are inferior to 0.05 (in bold), as well as predictive metric (Q2) is higher than 0.5. Variable importance in projection (VIP) is exploitable if and only if the predictive model is significant. Only VIP superior to 1 has been counted (VIP > 1 column).*

**TABLE 4:** Supervised multivariate PLS-DA models computed from metabolites found in blood serum metabolome differentiating two groups.

Model	R2X	R2Y	Q2	pR2Y	pQ2	VIP >1
Control vs. 3-h	0.472	0.989	0.877	**0.02**	**0.01**	120
3-h vs. 2-days	0.449	0.989	0.858	**0.01**	**0.01**	106
2-days vs. 1-week	0.269	0.901	0.426	0.90	0.60	110
1-week vs. Control	0.182	0.960	0.368	0.80	0.05	111
Control vs. 2-days	0.315	0.898	0.392	0.15	0.15	115
3-h vs. 1-week	0.452	0.990	0.859	**0.05**	**0.05**	117

*Model is considered significant if permutation metrics (pR2Y and pQ2, corresponding to the p-value of the permutation test) are inferior to 0.05 (in bold), as well as predictive metric (Q2) is higher than 0.5. Variable importance in projection (VIP) is exploitable if and only if the predictive model is significant. Only VIP superior to 1 has been counted (VIP > 1 column).*

**TABLE 5:** Supervised multivariate PLS-DA models computed from metabolites found in urine metabolome differentiating two groups.

Model	R2X	R2Y	Q2	pR2Y	pQ2	VIP >1
Control vs. 3-h	0.603	0.905	0.286	1.00	0.80	53
3-h vs. 2-days	0.626	0.833	0.423	0.35	0.10	55
2-days vs. 1-week	0.612	0.910	−0.061	0.05	0.50	43
1-week vs. Control	0.706	0.981	0.532	0.05	0.25	40
Control vs. 2-days	0.659	0.969	-0.537	0.30	0.95	40
3-h vs. 1-week	0.645	0.897	0.719	**0.05**	**0.05**	48

*Model is considered significant if permutation metrics (pR2Y and pQ2, corresponding to the p-value of the permutation test) are inferior to 0.05 (in bold), as well as predictive metric (Q2) is higher than 0.5. Variable importance in projection (VIP) is exploitable if and only if the predictive model is significant. Only VIP superior to 1 has been counted (VIP > 1 column).*

### Pathway Analyses on Cerebral and Peripheral Metabolomes Reveal Several Metabolic Dysregulations Over Time

#### Striatum Tissue

**FIGURE 7:**
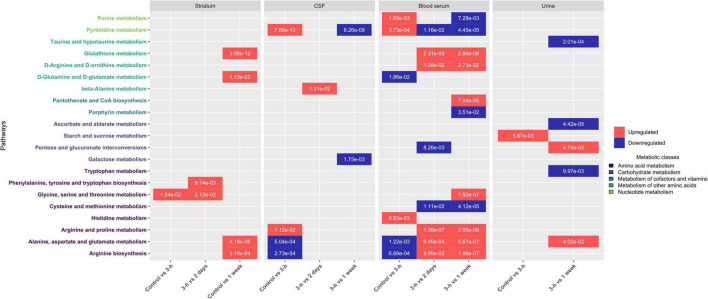
Impacted pathways in cerebral and peripheral metabolomes after acoustically-mediated BBB opening (BBBO). The adjusted *p*-value is written into tiles. Dysregulation is represented in red and blue, respectively for upregulation and downregulation of the considered pathway. Pathway names are coded with colors depending on their metabolic classes.

Taking the temporal dimension into consideration, the two first components of the PCA explained 33.5% of the total variance (Figure 3). Control and 3-h post-BBBO groups would share the same metabolic profile, while 2-days and 1-week post-BBBO groups would share another one. The analysis of other principal component axes (i.e., 2nd and 3rd) confirmed this metabolic profile sharing (data not shown). It was noted that the samples of the 2-days group are more heterogeneous than the other groups. Nevertheless, these two clusters of experimental groups appear to have different metabolic profiles. No supervised model using the PLS-DA method has been significantly predictive (Table 2). The statistical analysis of the intensities of metabolites identified a few dysregulated metabolic pathways in the hours and days following the BBB opening (Figure 7). Indeed, upregulation of glycine, serine, and threonine metabolism was observed between control and 3-h post-BBBO groups, but also between 3-h post-BBBO and 2-days post-BBBO groups. In addition, phenylalanine, tyrosine, and tryptophan biosynthesis were also upregulated between 3-h and 2-days post-BBBO groups. Finally, the most important metabolic perturbations in the striatum were observed between the control and 1-week post-BBBO groups, where glutathione metabolism, arginine biosynthesis, alanine, aspartate, and glutamate metabolism, and D-glutamine/D-glutamate metabolism, were significantly upregulated.

#### Cerebrospinal Fluid

As shown in Figure 4, PCA on CSF metabolites explained 27.3% of the total variance on the first two components. The CSF metabolome profile shifts radically 3 h after the acoustically-mediated BBBO compared to the control condition. Then, the CSF metabolome at 2 days and 1-week post-BBBO seem to recover and shared a similar metabolite profile to the control condition. Supervised PLS-DA analyses identified two significantly predictive models, thus confirming the unsupervised PCA findings (Table 3). Indeed, significant predictive models were identified when comparing control with 3-h post-BBBO groups and that of 3-h with 2-days post-BBBO groups. Then, univariate analyses were performed on CSF metabolites. Significantly different metabolites have been used to perform a metabolic pathway analysis as a function of post-BBBO time (Figure 7). The comparison of metabolic pathways between control and 3-h post-BBBO groups showed an upregulation of pyrimidine metabolism, arginine, and proline metabolisms, associated with a downregulation of arginine biosynthesis as well as alanine, aspartate, and glutamate metabolism. Then, an upregulation of beta-alanine metabolism occurred 2-days after BBBO. Finally, the comparative analysis of the results between 3-h and 1-week post-BBBO groups revealed the downregulation of galactose and pyrimidine metabolisms.

#### Blood Serum

Considering the blood serum samples of our four experimental groups, the two first components of the PCA explained 31.3% of the variance (Figure 5). On the score plot of this PCA, the blood serum metabolome profile shifts totally 3 h after BBBO compared to the control group. This metabolic profile undergoes another perturbation 2 days post-BBBO in comparison with the control group. Control and 1-week groups share the same metabolic profile. Supervised PLS-DA analyses showed three significant predictive models: control vs. 3-h post-BBBO group; 3-h post-BBBO group vs. 2-days post-BBBO group; 3-h post-BBBO group vs. 1-week post-BBBO group (Table 4). Univariate analysis of metabolite intensities in the blood serum allows determining of several significantly disrupt metabolites by the acoustically-mediated BBBO. Based on these significantly dysregulated metabolites, metabolic pathways analysis was performed and revealed that blood serum metabolome was the most disturbed biological matrices (Figure 7). The first perturbation of the blood serum metabolome was observed 3 h after BBBO in comparison with the control group. Indeed, metabolisms of purine, pyrimidine, and histidine were significantly upregulated while alanine, aspartate, and glutamate, as well as D-glutamate/D-glutamine metabolism and arginine biosynthesis were downregulated. A second metabolic perturbation was noticed between 2 days post-BBBO and 3-h post-BBBO group. Indeed, glutathione, arginine and proline, alanine, aspartate, and glutamate metabolisms, as well as arginine biosynthesis and D-arginine and D-ornithine metabolism were upregulated, while metabolisms of pyrimidine, cysteine, and methionine along with pentose/glucuronate interconversion were downregulated. Finally, a comparative analysis of metabolic pathways between 3-h and 1-week post-BBBO groups revealed the downregulation of purine and pyrimidine metabolisms but also porphyrin metabolism, cysteine, and methionine metabolism. This comparison also highlighted the upregulation of glutathione, D-arginine and D-ornithine, glycine, serine and threonine, arginine and proline, alanine, aspartate and glutamate metabolisms, and biosynthesis of pantothenate and CoA.

#### Urine

Data on urine samples seem to be more heterogenous than other matrices. As shown in Figure 6, a shift of urine metabolic profile between 3-h post-BBBO group and a cluster constituted by control, 2-days, and 1-week groups was identified. By analyzing the three first components in the score plot of PCA (data not shown), these three experimental groups were always clustered together, suggesting that these groups would share the same metabolic profile. Supervised multivariate analyses showed only one significant predictive model: 3-h post-BBBO group vs. 1-week group (Table 5). However, univariate data analyses on the level of urine metabolites identified some disturbed metabolic pathways (Figure 7). An upregulation of starch and sucrose metabolism was only observed between control and 3-h post-BBBO groups. Finally, several metabolic pathways were dysregulated between 3 h and 1 week after acoustically-mediated BBBO. Thus, the upregulated ones were alanine, aspartate, glutamate metabolism, and pentose/glucuronate interconversions. In addition, tryptophan metabolism, ascorbate, and aldarate metabolism as well as taurine and hypotaurine metabolism were significantly downregulated.

### Tryptophan Derivate Dosage Reveals Transient Changes in Biological Matrices

Tryptophan (TRP) is the precursor of biosynthesis pathways of serotonin (5-HT), kynurenine (KYN), and indole derivates. TRP and its metabolites are strongly involved in the kynurenine pathways (KP), which play a key role in the modulation of neurotransmission and neuroinflammation, thus influencing the physiology of the CNS (Cervenka et al., 2017). Taking it into consideration, TRP metabolism was explored in the three matrices including the striatum, CSF, and blood serum using HPLC-MS (Tables 6, 7). The analysis of TRP metabolism in the striatum showed that only the concentration of 5-HT decreased up to 2 days after BBBO (57.3 ± 14.6 nmol/g) and returned to normal level 1 week post-BBBO (63.9 ± 16.1 nmol/g). In addition, the 5-hydroxy indoleacetic acid (5-HIAA) is the single TRP metabolite in CSF whose the concentration significantly increased up to 2 days (0.739 ± 0.147 μM) after the BBBO, then returned to the control concentration (0.593 ± 0.122 μM) 1-week post-BBBO (Table 6). As expected, changes in TRP metabolism were more pronounced in the blood serum than in the other matrices (Table 7). In fact, 3-hydroxykynurenic (3-HK) concentration significantly decreased between 3-h (34.5 ± 16.2 nM) and 1 week (16.6 ± 0.59 nM) after BBBO. In addition, quinolinic acid (QUIN) concentration was significantly enhanced between the control group (0.7 ± 0.33 μM) and 3-h (2.17 ± 1.27 μM) post-BBBO group, but its concentration significantly decreased 1 week after BBBO (0.76 ± 0.38 μM) meaning the QUIN returns to its normal level 1 week post-BBBO in the blood serum. The dosage of TRP metabolite revealed an increasing trend in kynurenic acid (KYNA) level 3 h post-BBBO but then a significant decrease is observed between 3-h (0.31 ± 0.13 μM) and 2-days (0.14 ± 0.4 μM) after BBBO and finally a stabilization. Furthermore, a significant reduction in indole-3-acetic acid concentration was noticed between control (1.77 ± 0.76 μM) and 3-h (0.97 ± 0.36 μM) post-BBBO groups, while the indole-3-lactic acid concentration significantly decreased between 3-h (0.81 ± 0.3 μM) and 2-days (0.43 ± 0.19 μM) post-BBBO, then increased 1 week (0.46 ± 0.17 μM) after BBBO. We have to highlight that these observed modifications return to normal levels within 1 week after the BBBO.

**TABLE 6:** Concentrations of tryptophan derivates in CSF samples using targeted HPLC-MS method.

Metabolites	Control	3-h	2-days	1-week	Significant
Quinolinic acid	0.021	0.023	0.017	0.015	n.s.
	±0.008	±0.007	±0.006	±0.005	
Serotonin	0.753	0.798	0.71	0.711	n.s.
	±0.143	±0.212	±0.141	±0.079	
Kynurenine	0.051	0.058	0.053	0.045	n.s.
	±0.012	±0.016	±0.009	±0.010	
Tryptophan	1.285	1.786	1.684	1.734	n.s.
	±0.335	±0.472	±0.452	±0.277	
5-hydroxyindoleacetic acid	0.499	0.535	0.739	0.593	*Control vs. 2-days *3-h vs. 2-days
	±0.104	±0.071	±0.147	±0.122	

Standard deviations are expressed as a mean value; n.s. means not significant for non-parametric pairwise multiple comparisons after Bonferroni p-value adjustment; *<0.05; Groups showing significant differences are written (e.g., control vs 3-h). Concentrations are in micromolar (μM).

**TABLE 7:** Concentrations of tryptophan derivates in blood serum samples using targeted HPLC-MS method.

Metabolites	Control	3-h	2-days	1-week	Significant
Tryptophan	8.93 × 10^2^	1.18 × 10^3^	1.05 × 10^3^	9.30 × 10^2^	n.s.
	±4.06 × 10^2^	±4.47 × 10^2^	±2.40 × 10^2^	±2.52 × 10^2^	
Serotonin	5.77	6.82	7.19	6.73	n.s.
	±2.84	±1.98	±1.81	±2.67	
Kynurenine	6.10	6.91	5.77	4.91	n.s.
	±2.28	±2.64	±2.32	±1.15	
3-hydroxykynurenine	2.55 × 10^–2^	3.45 × 10^–2^	1.81 × 10^–2^	1.66 × 10^–2^	*3-h vs 1 week
	±1.91 × 10^–2^	±1.62 × 10^–2^	±8.01 × 10^–3^	±5.94 × 10^–3^	
Kynurenic acid	2.41 × 10^–1^	3.08 × 10^–1^	1.48 × 10^–1^	1.76 × 10^–1^	*3-h vs 2-days
	±1.16 × 10^–1^	±1.33 × 10^–1^	±4.04 × 10^–2^	±6.91 × 10^–2^	
Quinolinic acid	7.07 × 10^–1^	2.17	1.07	7.65 × 10^–1^	*Control vs 3-h *3-h vs 1-week
	±3.30 × 10^–1^	±1.27	±6.52 × 10^–1^	±3.79 × 10^–1^	
Indole-3-lactic acid	5.57 × 10^–1^	8.17 × 10^–1^	4.28 × 10^–1^	4.65 × 10^–1^	*3-h vs 2-days *3-h vs 1-week
	±2.74 × 10^–1^	±2.98 × 10^–1^	±1.58 × 10^–1^	±1.68 × 10^–1^	
Indole-3-acetic acid	1.77	9.71 × 10^–1^	1.49	1.17	*Control vs 3-h
	±7.64 × 10^–1^	±3.59 × 10^–1^	±5.20 × 10^–1^	±3.03 × 10^–1^	
5-hydroxyindoleacetic acid	2.14 × 10^–1^	2.59 × 10^–1^	1.99 × 10^–1^	2.05 × 10^–1^	n.s.
	±9.17 × 10^–2^	±8.29 × 10^–2^	±4.94 × 10^–2^	±5.57 × 10^–2^	

Standard deviations are specified under mean value; n.s. means not significant for non-parametric pairwise multiple comparisons after Bonferroni p-value adjustment; * < 0.05. Groups showing significant differences are writing (e.g., control vs. 3-h). Concentrations are in micromolar (μM).

## Discussion

The present study aimed to unravel the metabolic events following BBBO using MB-assisted US by answering the following questions: (1) How can acoustically-mediated BBBO processes trigger a global metabolic perturbation in the brain tissue and body fluids, such as the cerebrospinal fluid, the blood, and urine following BBBO? (2) Can it induce neuroinflammation? and (3) Can it potentially lead to an impairment in the neurotransmission? To achieve these goals, we performed a well-established analytical workflow using ^1^H NMR spectroscopy and HPLC-MS to assess metabolite levels and quantify tryptophan derivates involved in inflammatory and neurotransmission processes after BBBO.

### Metabolism of Central, Peri-Cerebral, and Peripheral Compartments

The effects of acoustically-mediated BBBO on brain metabolism were mainly investigated using fluorodeoxyglucose ([^18^F]FDG) PET/CT scan *via* the metabolism of intracerebral glucose (Yang et al., 2014; Horodyckid et al., 2017; Arif et al., 2020). Yang et al. (2014) described a reduction in [^18^F]FDG uptake in sonicated brain tissues in rats immediately and 1 week after BBBO compared to rodents not exposed to US, suggesting a decrease in the metabolism of cerebral energy supply. In contrast to these findings, our metabolomic data did not show any significant change in glucose metabolism after BBBO, indicating alternative recruitment of energetic resources. Our findings are supported by Horodyckid et al. (2017) who reported an absence of significant change in glucose metabolism in NHP 14 days after BBBO.

Importantly, all these functional imaging studies only focused on energetic metabolism in the cerebral compartment. In the present study, the metabolic signature after acoustically-mediated BBBO is reported in the cerebral compartment, but also in the peripheral zones, which, to the best of our knowledge, has never been shown before. Based on PCA analyses (Figures 3–6) and the identification of dysregulated metabolic pathways (Figure 7), metabolic perturbations are visible in both central and peripheral matrices. However, these changes in the metabolic signature of the brain, CSF, blood serum, and urine metabolomes show specific and sequential kinetics. Indeed, our multivariate data reveal that metabolic perturbations occur in the blood serum metabolome up to 2 days after BBBO with a peak at 3 h. In the CSF, the peak of metabolic dysregulation occurs 3 h post-BBBO. Metabolic perturbations in the brain metabolome and in the urine metabolome are more latent and the perturbation peak occurs 1 week post-BBBO. These data could be explained by the rapid CSF turnover. Indeed, the CSF is renewed around 12–13 times a day, which could rapidly wipe the metabolic perturbation (Simon and Iliff, 2016). In addition, the delayed response of brain tissue could be associated with the setup of compensatory mechanisms that could hide for a while the metabolic response (Hylin et al., 2017). By analyzing metabolic pathways, data showed that arginine, proline, alanine, aspartate, and glutamate metabolisms are mainly and significantly disrupted in all matrices. All these metabolic pathways are interconnected with each other. It is known that alanine, aspartate, asparagine, glutamate, and glutamine are input metabolites for arginine-associated metabolic processes, i.e., urea cycle, tricarboxylic acid cycle, and nitrogen metabolism. Arginine plays a major role in the energetic cell supply and homeostasis by regulating nitrogen levels and immune processes (Tong and Barbul, 2004). This amino acid is also the immediate precursor of nitric oxide (NO) responsible for the vasodilation of blood vessels (Wiesinger, 2001). Indeed, Raymond et al. (2007) reported short-lived vasoconstriction of the blood vessel right after sonication. Thus, NO secretion could occur in response to BBBO vasomotor effects. In addition, several metabolic pathways, namely arginine and glutamate metabolism, involved in energetic metabolism were disrupted in the blood serum and CSF metabolomes immediately after the BBBO, but also in the brain metabolome, 1 week later. Despite BBB resealing, 1 h after US exposure (Supplementary Figure S1), this study evidences that metabolic pathways in the brain, blood serum, and urine metabolomes were durably modified more than a week after BBBO. Further exploration of time points greater than a week after BBBO is warranted to fully ascertain the time to complete recovery, i.e., a metabolome fingerprint similar to that in the physiological state. Altogether, these results indicate that acoustically-mediated BBBO induces significant metabolic changes, not only in the brain tissue but also in the CSF, blood serum, and urine. Based on these multi-compartment data that show more durable effects in comparison to the standard arsenal of readout modalities, this study warrants further application of this sensitive and powerful metabolomic readout in acoustically-mediated BBBO investigations.

By comparing the kinetic of blood serum metabolome perturbations and the number of dysregulated metabolisms within the brain, CSF, and urine metabolomes, the blood serum metabolome was shown to be the most impacted matrix after acoustically-mediated BBBO. These metabolic data are consistent with recent available literature evidencing the effects of MB-assisted US on endothelial cells, thus reporting MB-assisted US-induced bioeffects in the cerebrovascular system, mainly. Indeed, Sheikov et al. (2004) explored the biophysical mechanisms involved in acoustically-mediated BBBO on brain endothelial cells using electron microscopy techniques and reported several cytoarchitectural changes including the disruption of TJs, the formations of intercellular cleft, and the fenestration on endothelial cells. In addition, exposure of the endothelial wall to acoustically-mediated mechanical stress might induce the secretion of pro-inflammatory agents and/or biologically active molecules by the endothelium or perivascular environment, thus leading to the degradation of TJ proteins and, ultimately, BBB alteration (Cucullo et al., 2003; Brown et al., 2016). Indeed, the metabolic changes observed in our study could be attributed to intra- and inter-cellular changes at the endothelial cell level that were induced by the MB-assisted US. In this, our data support the link between the biophysical effects reported in the available literature and the metabolic changes observed in our study.

### Neuroinflammation

Using PET imaging and transcriptomics, previous preclinical studies described conflicting results regarding neuroinflammation processes induced by acoustically-mediated BBBO. Sinharay et al. (2019) observed an increase in the binding of [^18^F]DPA-714 (a biomarker of translocator protein involved in inflammatory processes) in sonicated brain regions compared to controls, regardless of the number of sonications (i.e., 1–6) and the duration after BBBO (i.e., 24 h or 10 days). Besides, transcriptomics studies of brain tissues showed overexpression of genes associated with cytokine biosynthesis, thus demonstrating that acoustically-mediated BBBO induces neuroinflammation. This inflammation may participate in the induction and/or maintenance of BBB permeability and the resulting metabolic changes (Kovacs et al., 2017; McMahon and Hynynen, 2017; McMahon et al., 2017, 2020).

To explore further tissue inflammation after acoustically-mediated BBBO, a previously described targeted metabolomic approach using HPLC-MS was applied (Lefevre et al., 2019; Alarcan et al., 2021). We dosed the TRP derivates, especially metabolites from the KP cascade in the brain tissue, in the CSF, and in the blood serum. KP produces several neuroactive metabolites, which are synthetized following inflammatory events (Tanaka et al., 2021). In the CSF, QUIN is synthesized by microglia or infiltrated peripheral macrophages, while KYNA is produced by astrocytes. In the periphery, the major organ where the synthesis of kynurenines occur is the liver (Torok et al., 2020). 3-HK and 3-hydroxyanthranilic acid (3-HAA) are well known to induce oxidative stress and apoptosis (Cervenka et al., 2017). QUIN results in neurotoxicity by inducing excitotoxicity, free-radical formation, mitochondrial dysfunction, apoptosis or necrosis, and cytoskeletal destabilization (el-Defrawy et al., 1986). As neuroprotective compounds, picolinic acid (PIC) and KYNA inhibit excitotoxicity and modulate the immune response, respectively (Tanaka et al., 2021). TRP, 3-HK, and KYN can reach the CNS through the BBB, whereas QUIN and KYNA do not cross the BBB (Fukui et al., 1991). Our metabolomics results showed a significant increase in QUIN concentration in the blood serum metabolome 3 h after BBBO and a significant decrease in KYNA and 3-HK levels between 3-h and 2-days post-BBBO groups. These metabolic perturbations were only found in the blood serum and not in other biological matrices (i.e., brain and CSF). These results could be ascribed (i) either to the production of these metabolites in the liver consecutively to an inflammatory injury or a classic endocrine response to stress, (ii) or to neuroinflammation and, therefore, a passage of the metabolites from the brain tissue to blood compartment through the opened BBB, or (iii) to a vascular inflammation caused by the mechanical action of oscillating MBs on the endothelial wall. This last explanation is supported by McMahon et al. (2017) who performed a transcriptomics analysis from 6 to 24 h after acoustically-mediated BBBO only on vascular endothelial cells after acoustically-mediated BBBO (McMahon and Hynynen, 2017). These authors showed significant overexpression of proinflammatory and antiapoptotic-associated genes, suggesting the initiation of vascular inflammation. This upregulation of gene expression implies the activation and/or repression of the genetic machinery, which requires the biosynthesis of metabolites, specifically nucleotides as reported in our metabolomics analysis. Indeed, this study pinpoints an upregulation of nucleotide metabolisms (i.e., purine and pyrimidine metabolisms) up to 3 h post-BBBO in the blood serum. These metabolisms return to a basal state 1 week post-BBBO. In addition, these transcriptomics data and our metabolomics results (Figure 7) are supported by Brown et al. (2016) who investigated the metabolic consequences of the exposure of an organ-on-a-chip model of the human neurovascular unit to a pro-inflammatory stimulus, defined as a cytokine cocktail. The exposure of this stimulus to the vascular compartment induced a significant increase in BBB permeability by disrupting TJs. Their metabolomics analysis revealed a significant perturbation in aspartate and asparagine, and glycine, serine, alanine, and threonine metabolisms, but also significant changes in valine, leucine, and isoleucine, tryptophan, tyrosine, and lysine metabolisms in both vascular and brain compartments. Besides, cytokine stimulus impacted the pyrimidine metabolism only in the vascular compartment. Their metabolic pathways analysis also revealed significant disruption of coenzyme A biosynthesis from pantothenate, and methionine and cysteine metabolism on the vascular side. Most of these disrupted metabolic pathways are also observed in our metabolomic data in central and peripheral metabolomes.

Altogether, these results suggest that the acoustically-mediated BBBO could induce vascular inflammation. In addition to the acoustically-mediated mechanical stress on the endothelial wall, this inflammation may contribute to the metabolic changes in the blood serum but also in other biological matrices. In our study, the absence of neuroinflammation may be explained by the lower acoustic regime applied, i.e., acoustic parameters, dose, and type of MBs, mainly, in comparison to those in previous preclinical studies (Kovacs et al., 2017; McMahon and Hynynen, 2017; McMahon et al., 2017; Sinharay et al., 2019). In our opinion, further metabolomic investigations exploring catecholaminergic derivates, but also arachidonic acid derivates (Wang et al., 2021) would provide key insights into the influence of the US regime and MB usage on inflammatory processes in the central (in the ipsilateral target and in its contralateral region) and peripheral compartments. These studies would provide highly detailed information on the transcriptomics profiles subsequent to BBBO, as also initiated by McMahon and Hynynen (2017) and Kovacs et al. (2017, 2018) who clearly demonstrated that BBBO-induced inflammatory response is related to the dose of MBs; it was well-established that the bioeffects of the BBBO directly depend on BBBO regime and MB type and injection settings, such as infusion type and rate, i.e., bolus or slow injection, dose, and initial concentration. These results could be compared to complementary IHC studies using inflammation and BBB biomarkers.

### Neurotransmission

To the best of our knowledge, the effects of acoustically-mediated BBBO on neurotransmission have never been investigated; this study explored these bioeffects by dosing 5-HT and its derivates originating from the TRP metabolism by a targeted HPLC-MS approach (Lefevre et al., 2019; Alarcan et al., 2021). Indeed, the striatum is involved in dopaminergic, cholinergic, and GABAergic neurotransmissions. In addition, this functional region is widely innervated by the projections of serotoninergic neurons from the dorsal raphe nucleus that are localized in the brain stem (Charnay and Leger, 2010; Nakamura, 2013). This nerve pathway is involved in the regulation of limbic processes such as behavior and mood, especially in stress response and stress-associated disorders (Waselus et al., 2006). TRP dosage in the striatum showed a significant decrease in serotonin concentration between the control and 2 days post-BBBO groups. In addition, a significant increase in the concentration of main serotonin catabolite, i.e., 5-HIAA, was detected in the CSF between 3-h and 2 days post-BBBO groups, then its concentration decreased between 2-days and 1-week groups. Of note, no significant change of these 5-HT derivates was found in the blood serum, suggesting that these variations only occur in the central compartment and are not due to peripheral gastrointestinal 5-HT metabolism (Stasi et al., 2019). These results demonstrated an upregulation of the serotonin catabolism in our experimental conditions, thus bringing the first evidence that acoustically-mediated BBBO could disrupt serotonergic neurotransmission. As previously described with regards to tissue inflammation, our study warrants a wider use of metabolomics to explore the influence of US- and MB-related parameters on serotonergic neurotransmission. These results could be confirmed by further PET/CT studies using radiotracers dedicated to the investigation of serotonergic neurotransmission (Buchecker et al., 2020; Colom et al., 2021). In the present study, only the serotonergic neurotransmission was investigated while the striatum is also involved in GABAergic and dopaminergic neurotransmission. Hence, additional metabolomics investigations are warranted to assess the potential effects of acoustically-mediated BBBO on these specific neurotransmission pathways, but also those operating in other brain regions.

## Conclusion

In conclusion, our multimodal and multimatrix metabolomic investigations provided the first metabolic signature related to the acoustically-mediated BBBO within the striatum tissue and its surrounding compartments. The stronger metabolic response to the acoustically-mediated BBBO was observed in the brain vascular compartment with especially the potential presence of vascular inflammation, but also metabolic modifications of serotonergic neurotransmission pathways in the striatum. These discrepancies in metabolic profiles in all biological matrices were present up to 1 week following BBBO, but no sign of discomfort could be observed. Nevertheless, further investigations are warranted to extensively evaluate the influence of the US and MB settings on brain metabolism and neurological processes such as neuroinflammation and neurotransmission.

## Data Availability Statement

The datasets presented in this study can be found in online repositories. The names of the repository/repositories and accession number(s) can be found below: MetaboLights, accession number: MTBLS4423.

## Ethics Statement

The animal study was reviewed and approved by the Animal Care and Regional Committee for Ethics in Animal Experiments, Centre Val-de-Loire (2018011016593068) in accordance with European Directive 2010/63/EU for animal experiments.

## Author Contributions

AP, J-ME, and LN-D designed the experiments and wrote the manuscript. AP, EO, and SB performed the *in vivo* experiments. AM, AP, and CD prepared the biological tissue and fluids for mass spectroscopy (MS) and nuclear magnetic resonance (NMR) spectroscopy. AL and CD took care of the development of MS targeted method and the MS acquisition of samples. TI provided the technical support. AP analyzed the metabolomic data. AB and PE reviewed the manuscript. All authors contributed to the article and approved the submitted version.

## Conflict of Interest

The authors declare that the research was conducted in the absence of any commercial or financial relationships that could be construed as a potential conflict of interest.

## Publisher’s Note

All claims expressed in this article are solely those of the authors and do not necessarily represent those of their affiliated organizations, or those of the publisher, the editors and the reviewers. Any product that may be evaluated in this article, or claim that may be made by its manufacturer, is not guaranteed or endorsed by the publisher.

## References

[B1] Abbott N. J. Patabendige A. A. Dolman D. E. Yusof S. R. Begley D. J. (2010). Structure and function of the blood-brain barrier. *Neurobiol. Dis.* 37 13–25. 10.1016/j.nbd.2009.07.030 19664713

[B2] Alarcan H. Chaumond R. Emond P. Benz-De Bretagne I. Lefevre A. Bakkouche S. E. (2021). Some CSF kynurenine pathway intermediates associated with disease evolution in amyotrophic lateral sclerosis. *Biomolecules* 11:691. 10.3390/biom11050691 34063031 PMC8147980

[B3] Alonso A. Reinz E. Fatar M. Hennerici M. G. Meairs S. (2011). Clearance of albumin following ultrasound-induced blood-brain barrier opening is mediated by glial but not neuronal cells. *Brain Res.* 1411 9–16. 10.1016/j.brainres.2011.07.006 21820103

[B4] Alshammari T. M. Al-Hassan A. A. Hadda T. B. Aljofan M. (2015). Comparison of different serum sample extraction methods and their suitability for mass spectrometry analysis. *Saudi Pharm. J.* 23 689–697. 10.1016/j.jsps.2015.01.023 26702265 PMC4669428

[B5] Arif W. M. Elsinga P. H. Gasca-Salas C. Versluis M. Martinez-Fernandez R. Dierckx R. (2020). Focused ultrasound for opening blood-brain barrier and drug delivery monitored with positron emission tomography. *J. Control Release* 324 303–316. 10.1016/j.jconrel.2020.05.020 32428519

[B6] Barar J. Rafi M. A. Pourseif M. M. Omidi Y. (2016). Blood-brain barrier transport machineries and targeted therapy of brain diseases. *Bioimpacts* 6 225–248. 10.15171/bi.2016.30 28265539 PMC5326671

[B7] Baseri B. Choi J. J. Tung Y. S. Konofagou E. E. (2010). Multi-modality safety assessment of blood-brain barrier opening using focused ultrasound and definity microbubbles: a short-term study. *Ultrasound Med. Biol.* 36 1445–1459. 10.1016/j.ultrasmedbio.2010.06.005 20800172 PMC3968780

[B8] Blasco H. Corcia P. Pradat P. F. Bocca C. Gordon P. H. Veyrat-Durebex C. (2013). Metabolomics in cerebrospinal fluid of patients with amyotrophic lateral sclerosis: an untargeted approach via high-resolution mass spectrometry. *J. Proteome Res.* 12 3746–3754. 10.1021/pr400376e 23859630

[B9] Brown J. A. Codreanu S. G. Shi M. Sherrod S. D. Markov D. A. Neely M. D. (2016). Metabolic consequences of inflammatory disruption of the blood-brain barrier in an organ-on-chip model of the human neurovascular unit. *J. Neuroinflammation* 13:306. 10.1186/s12974-016-0760-y 27955696 PMC5153753

[B10] Buchecker V. Waldron A. M. van Dijk R. M. Koska I. Brendel M. von Ungern-Sternberg B. (2020). [(18)F]MPPF and [(18)F]FDG MuPET imaging in rats: impact of transport and restraint stress. *EJNMMI Res.* 10:112. 10.1186/s13550-020-00693-3 32990819 PMC7524912

[B11] Bulat M. Klarica M. (2011). Recent insights into a new hydrodynamics of the cerebrospinal fluid. *Brain Res. Rev.* 65 99–112. 10.1016/j.brainresrev.2010.08.002 20817024

[B12] Cervenka I. Agudelo L. Z. Ruas J. L. (2017). Kynurenines: tryptophan’s metabolites in exercise, inflammation, and mental health. *Science* 357:eaaf9794. 10.1126/science.aaf9794 28751584

[B13] Charnay Y. Leger L. (2010). Brain serotonergic circuitries. *Dialogues Clin. Neurosci.* 12 471–487. 10.31887/DCNS.2010.12.4/ycharnay 21319493 PMC3181988

[B14] Colom M. Vidal B. Fieux S. Redoute J. Costes N. Lavenne F. (2021). [(18)F]F13640, a 5-HT1A receptor radiopharmaceutical sensitive to brain serotonin fluctuations. *Front. Neurosci.* 15:622423. 10.3389/fnins.2021.622423 33762906 PMC7982540

[B15] Craig A. Cloarec O. Holmes E. Nicholson J. K. Lindon J. C. (2006). Scaling and normalization effects in NMR spectroscopic metabonomic data sets. *Anal. Chem.* 78 2262–2267. 10.1021/ac0519312 16579606

[B16] Cucullo L. Marchi N. Marroni M. Fazio V. Namura S. Janigro D. (2003). Blood-brain barrier damage induces release of Alpha2-macroglobulin. *Mol. Cell. Proteomics* 2 234–241. 10.1074/mcp.M200077-MCP200 12714567

[B17] Daneman R. Prat A. (2015). The blood-brain barrier. *Cold Spring Harb. Perspect. Biol.* 7:a020412. 10.1101/cshperspect.a020412 25561720 PMC4292164

[B18] Dieme B. Lefevre A. Nadal-Desbarats L. Galineau L. Madji Hounoum B. Montigny F. (2017). Workflow methodology for rat brain metabolome exploration using NMR, LC-MS and GC-MS analytical platforms. *J. Pharm. Biomed. Anal.* 142 270–278. 10.1016/j.jpba.2017.03.068 28531831

[B19] Dieme B. Mavel S. Blasco H. Tripi G. Bonnet-Brilhault F. Malvy J. (2015). Metabolomics study of urine in autism spectrum disorders using a multiplatform analytical methodology. *J. Proteome Res.* 14 5273–5282. 10.1021/acs.jproteome.5b00699 26538324

[B20] Dupuy C. Castelnau P. Mavel S. Lefevre A. Nadal-Desbarats L. Bodard S. (2021). SHR/NCRL rats as a model of ADHD can be discriminated from controls based on their brain, blood, or urine metabolomes. *Transl. Psychiatry* 11:235. 10.1038/s41398-021-01344-4 33888684 PMC8062531

[B21] el-Defrawy S. R. Boegman R. J. Jhamandas K. Beninger R. J. (1986). The neurotoxic actions of quinolinic acid in the central nervous system. *Can. J. Physiol. Pharmacol.* 64 369–375. 10.1139/y86-060 2939936

[B22] Escoffre J. M. Novell A. de Smet M. Bouakaz A. (2013). Focused ultrasound mediated drug delivery from temperature-sensitive liposomes: in-vitro characterization and validation. *Phys. Med. Biol.* 58 8135–8151. 10.1088/0031-9155/58/22/8135 24200816

[B23] Fukui S. Schwarcz R. Rapoport S. I. Takada Y. Smith Q. R. (1991). Blood-brain barrier transport of kynurenines: implications for brain synthesis and metabolism. *J. Neurochem.* 56 2007–2017. 10.1111/j.1471-4159.1991.tb03460.x 1827495

[B24] Gaillard A. Jaber M. (2011). Rewiring the brain with cell transplantation in Parkinson’s disease. *Trends Neurosci.* 34 124–133. 10.1016/j.tins.2011.01.003 21316770

[B25] Gerstenmayer M. Fellah B. Magnin R. Selingue E. Larrat B. (2018). Acoustic transmission factor through the rat skull as a function of body mass, frequency and position. *Ultrasound Med. Biol.* 44 2336–2344. 10.1016/j.ultrasmedbio.2018.06.005 30076032

[B26] Grahn J. A. Parkinson J. A. Owen A. M. (2008). The cognitive functions of the caudate nucleus. *Prog. Neurobiol.* 86 141–155. 10.1016/j.pneurobio.2008.09.004 18824075

[B27] Horodyckid C. Canney M. Vignot A. Boisgard R. Drier A. Huberfeld G. (2017). Safe long-term repeated disruption of the blood-brain barrier using an implantable ultrasound device: a multiparametric study in a primate model. *J. Neurosurg.* 126 1351–1361. 10.3171/2016.3.JNS151635 27285538

[B28] Hylin M. J. Kerr A. L. Holden R. (2017). Understanding the mechanisms of recovery and/or compensation following injury. *Neural Plast.* 2017:7125057. 10.1155/2017/7125057 28512585 PMC5415868

[B29] Ji R. Karakatsani M. E. Burgess M. Smith M. Murillo M. F. Konofagou E. E. (2021). Cavitation modulated inflammatory response following focused ultrasound blood-brain barrier opening. *J. Control Release* 337 458–471. 10.1016/j.jconrel.2021.07.042 34324895 PMC8440441

[B30] Kanehisa M. Furumichi M. Tanabe M. Sato Y. Morishima K. (2017). Kegg: new perspectives on genomes, pathways, diseases and drugs. *Nucleic Acids Res.* 45 D353–D361. 10.1093/nar/gkw1092 27899662 PMC5210567

[B31] Kim H. Park M. A. Wang S. Chiu A. Fischer K. Yoo S. S. (2013). PET/CT imaging evidence of FUS-mediated (18)F-FDG uptake changes in rat brain. *Med. Phys.* 40:033501. 10.1118/1.4789916 23464343 PMC3585755

[B32] Kim S. Chen J. Cheng T. Gindulyte A. He J. He S. (2021). Pubchem in 2021: new data content and improved web interfaces. *Nucleic Acids Res.* 49 D1388–D1395. 10.1093/nar/gkaa971 33151290 PMC7778930

[B33] Kovacs Z. I. Burks S. R. Frank J. A. (2018). Focused ultrasound with microbubbles induces sterile inflammatory response proportional to the blood brain barrier opening: attention to experimental conditions. *Theranostics* 8 2245–2248. 10.7150/thno.24181 29722362 PMC5928885

[B34] Kovacs Z. I. Kim S. Jikaria N. Qureshi F. Milo B. Lewis B. K. (2017). Disrupting the blood-brain barrier by focused ultrasound induces sterile inflammation. *Proc. Natl. Acad. Sci. U.S.A.* 114 E75–E84. 10.1073/pnas.1614777114 27994152 PMC5224365

[B35] Lefevre A. Mavel S. Nadal-Desbarats L. Galineau L. Attucci S. Dufour D. (2019). Validation of a global quantitative analysis methodology of tryptophan metabolites in mice using LC-MS. *Talanta* 195 593–598. 10.1016/j.talanta.2018.11.094 30625588

[B36] Lefort G. Liaubet L. Canlet C. Tardivel P. Pere M. C. Quesnel H. (2019). ASICS: an R package for a whole analysis workflow of 1D 1H NMR spectra. *Bioinformatics* 35 4356–4363. 10.1093/bioinformatics/btz248 30977816

[B37] Lex A. Gehlenborg N. Strobelt H. Vuillemot R. Pfister H. (2014). Upset: visualization of intersecting sets. *IEEE Trans. Vis. Comput. Graph.* 20 1983–1992. 10.1109/TVCG.2014.2346248 26356912 PMC4720993

[B38] Liu H. L. Wai Y. Y. Chen W. S. Chen J. C. Hsu P. H. Wu X. Y. (2008). Hemorrhage detection during focused-ultrasound induced blood-brain-barrier opening by using susceptibility-weighted magnetic resonance imaging. *Ultrasound Med. Biol.* 34 598–606. 10.1016/j.ultrasmedbio.2008.01.011 18313204

[B39] Mainprize T. Lipsman N. Huang Y. Meng Y. Bethune A. Ironside S. (2019). Blood-brain barrier opening in primary brain tumors with non-invasive MR-guided focused ultrasound: a clinical safety and feasibility study. *Sci. Rep.* 9:321. 10.1038/s41598-018-36340-0 30674905 PMC6344541

[B40] McDannold N. Vykhodtseva N. Raymond S. Jolesz F. A. Hynynen K. (2005). MRI-guided targeted blood-brain barrier disruption with focused ultrasound: histological findings in rabbits. *Ultrasound Med. Biol.* 31 1527–1537. 10.1016/j.ultrasmedbio.2005.07.010 16286030

[B41] McMahon D. Hynynen K. (2017). Acute inflammatory response following increased blood-brain barrier permeability induced by focused ultrasound is dependent on microbubble dose. *Theranostics* 7 3989–4000. 10.7150/thno.21630 29109793 PMC5667420

[B42] McMahon D. Bendayan R. Hynynen K. (2017). Acute effects of focused ultrasound-induced increases in blood-brain barrier permeability on rat microvascular transcriptome. *Sci. Rep.* 7:45657. 10.1038/srep45657 28374753 PMC5379491

[B43] McMahon D. Oakden W. Hynynen K. (2020). Investigating the effects of dexamethasone on blood-brain barrier permeability and inflammatory response following focused ultrasound and microbubble exposure. *Theranostics* 10 1604–1618. 10.7150/thno.40908 32042325 PMC6993222

[B44] McMahon D. Poon C. Hynynen K. (2019). Evaluating the safety profile of focused ultrasound and microbubble-mediated treatments to increase blood-brain barrier permeability. *Expert Opin. Drug Deliv.* 16 129–142. 10.1080/17425247.2019.1567490 30628455 PMC6576291

[B45] Nakamura K. (2013). The role of the dorsal raphe nucleus in reward-seeking behavior. *Front. Integr. Neurosci.* 7:60. 10.3389/fnint.2013.00060 23986662 PMC3753458

[B46] Olumolade O. O. Wang S. Samiotaki G. Konofagou E. E. (2016). Longitudinal motor and behavioral assessment of blood-brain barrier opening with transcranial focused ultrasound. *Ultrasound Med. Biol.* 42 2270–2282. 10.1016/j.ultrasmedbio.2016.05.004 27339763 PMC5381156

[B47] Pardridge W. M. Oldendorf W. H. Cancilla P. Frank H. J. (1986). Blood-brain barrier: interface between internal medicine and the brain. *Ann. Intern. Med.* 105 82–95. 10.7326/0003-4819-105-1-82 2872846

[B48] Presset A. Bonneau C. Kazuyoshi S. Nadal-Desbarats L. Mitsuyoshi T. Bouakaz A. (2020). Endothelial cells, first target of drug delivery using microbubble-assisted ultrasound. *Ultrasound Med. Biol.* 46 1565–1583. 10.1016/j.ultrasmedbio.2020.03.013 32331799

[B49] Psychogios N. Hau D. D. Peng J. Guo A. C. Mandal R. Bouatra S. (2011). The human serum metabolome. *PLoS One* 6:e16957. 10.1371/journal.pone.0016957 21359215 PMC3040193

[B50] Raymond S. B. Skoch J. Hynynen K. Bacskai B. J. (2007). Multiphoton imaging of Ultrasound/Optison mediated cerebrovascular effects in vivo. *J. Cereb. Blood Flow Metab.* 27 393–403. 10.1038/sj.jcbfm.9600336 16685254

[B51] Schober A. (2004). Classic toxin-induced animal models of Parkinson’s disease: 6-OHDA and MPTP. *Cell Tissue Res.* 318 215–224. 10.1007/s00441-004-0938-y 15503155

[B52] Segal M. B. (2000). The choroid plexuses and the barriers between the blood and the cerebrospinal fluid. *Cell. Mol. Neurobiol.* 20 183–196. 10.1023/a:1007045605751 10696509 PMC11537546

[B53] Sheikov N. McDannold N. Jolesz F. Zhang Y. Z. Tam K. Hynynen K. (2006). Brain arterioles show more active vesicular transport of blood-borne tracer molecules than capillaries and venules after focused ultrasound-evoked opening of the blood-brain barrier. *Ultrasound Med. Biol.* 32 1399–1409. 10.1016/j.ultrasmedbio.2006.05.015 16965980

[B54] Sheikov N. McDannold N. Sharma S. Hynynen K. (2008). Effect of focused ultrasound applied with an ultrasound contrast agent on the tight junctional integrity of the brain microvascular endothelium. *Ultrasound Med. Biol.* 34 1093–1104. 10.1016/j.ultrasmedbio.2007.12.015 18378064 PMC2518085

[B55] Sheikov N. McDannold N. Vykhodtseva N. Jolesz F. Hynynen K. (2004). Cellular mechanisms of the blood-brain barrier opening induced by ultrasound in presence of microbubbles. *Ultrasound Med. Biol.* 30 979–989. 10.1016/j.ultrasmedbio.2004.04.010 15313330

[B56] Simon M. J. Iliff J. J. (2016). Regulation of cerebrospinal fluid (CSF) flow in neurodegenerative, neurovascular and neuroinflammatory disease. *Biochim. Biophys. Acta* 1862 442–451. 10.1016/j.bbadis.2015.10.014 26499397 PMC4755861

[B57] Sinharay S. Tu T. W. Kovacs Z. I. Schreiber-Stainthorp W. Sundby M. Zhang X. (2019). In vivo imaging of sterile microglial activation in rat brain after disrupting the blood-brain barrier with pulsed focused ultrasound: [18f]DPA-714 PET study. *J. Neuroinflammation* 16:155. 10.1186/s12974-019-1543-z 31345243 PMC6657093

[B58] Stasi C. Sadalla S. Milani S. (2019). The relationship between the serotonin metabolism, gut-microbiota and the gut-brain axis. *Curr. Drug Metab.* 20 646–655. 10.2174/1389200220666190725115503 31345143

[B59] Tanaka M. Toth F. Polyak H. Szabo A. Mandi Y. Vecsei L. (2021). Immune influencers in action: metabolites and enzymes of the tryptophan-kynurenine metabolic pathway. *Biomedicines* 9:734. 10.3390/biomedicines9070734 34202246 PMC8301407

[B60] Thevenot E. A. Roux A. Xu Y. Ezan E. Junot C. (2015). Analysis of the human adult urinary metabolome variations with age, body mass index, and gender by implementing a comprehensive workflow for univariate and OPLS statistical analyses. *J. Proteome Res.* 14 3322–3335. 10.1021/acs.jproteome.5b00354 26088811

[B61] Tong B. C. Barbul A. (2004). Cellular and physiological effects of arginine. *Mini Rev. Med. Chem.* 4 823–832. 10.2174/1389557043403305 15544543

[B62] Torok N. Tanaka M. Vecsei L. (2020). Searching for peripheral biomarkers in neurodegenerative diseases: the tryptophan-kynurenine metabolic pathway. *Int. J. Mol. Sci.* 21:9338. 10.3390/ijms21249338 33302404 PMC7762583

[B63] Tsai H. C. Chen W. S. Inserra C. Wei K. C. Liu H. L. (2018). Safety evaluation of frequent application of microbubble-enhanced focused ultrasound blood-brain-barrier opening. *Sci. Rep.* 8:17720. 10.1038/s41598-018-35677-w 30531863 PMC6286368

[B64] Wang B. Wu L. Chen J. Dong L. Chen C. Wen Z. (2021). Metabolism pathways of arachidonic acids: mechanisms and potential therapeutic targets. *Signal Transduct. Target Ther.* 6:94. 10.1038/s41392-020-00443-w 33637672 PMC7910446

[B65] Waselus M. Galvez J. P. Valentino R. J. Van Bockstaele E. J. (2006). Differential projections of dorsal raphe nucleus neurons to the lateral septum and striatum. *J. Chem. Neuroanat.* 31 233–242. 10.1016/j.jchemneu.2006.01.007 16540283

[B66] Wiesinger H. (2001). Arginine metabolism and the synthesis of nitric oxide in the nervous system. *Prog. Neurobiol.* 64 365–391. 10.1016/s0301-0082(00)00056-3 11275358

[B67] Wishart D. S. Feunang Y. D. Marcu A. Guo A. C. Liang K. Vazquez-Fresno R. (2018). Hmdb 4.0: the human metabolome database for 2018. *Nucleic Acids Res.* 46 D608–D617. 10.1093/nar/gkx1089 29140435 PMC5753273

[B68] Yang F. Y. Chang W. Y. Chen J. C. Lee L. C. Hung Y. S. (2014). Quantitative assessment of cerebral glucose metabolic rates after blood-brain barrier disruption induced by focused ultrasound using FDG-MicroPET. *Neuroimage* 90 93–98. 10.1016/j.neuroimage.2013.12.033 24368263

