# Supplementary figures and images for: First Metabolomic Signature of Blood-Brain Barrier Opening Induced by Microbubble-Assisted Ultrasound
Source: Front Mol Neurosci. 2022 Jun 20;15:888318. doi: 10.3389/fnmol.2022.888318 (PMC9251546; doi:10.3389/fnmol.2022.888318)

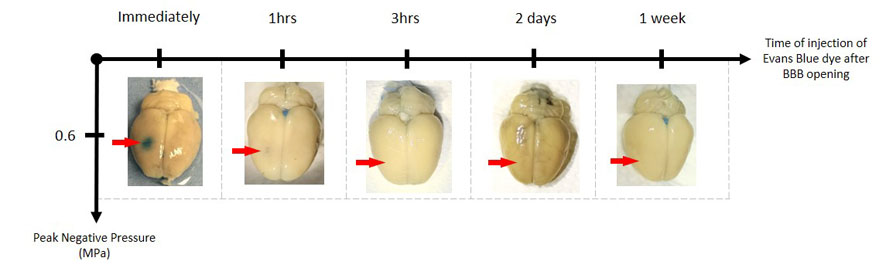

Supplement: Supplementary file 1 [file Image_1.JPEG]
