# Supplementary material for: First Metabolomic Signature of Blood-Brain Barrier Opening Induced by Microbubble-Assisted Ultrasound
Source: Front Mol Neurosci. 2022 Jun 20;15:888318. doi: 10.3389/fnmol.2022.888318 (PMC9251546; doi:10.3389/fnmol.2022.888318)
Supplement: Supplementary file 2 [file Data_Sheet_1.docx]

Supplementary Material

**Table S1:** Metabolites showing significantly different intensities by group-to-group univariate comparisons found in striatum 3hrs, 2 days, and 1 week after acoustically-mediated BBB opening. ** < 0.05, ** < 0.01* and **** 0.001.*

| Metabolites | Groups | | Adjusted p-value | Signif. | Log2(FC) |
| --- | --- | --- | --- | --- | --- |
| 2-Deoxyguanosine | Control | 1 week | 0.012 | * | 1.56 |
| 2-Propanol | Control | 1 week | 0.033 | * | 2.51 |
| 3-Methylhistamine | Control | 2 days | 0.028 | * | -0.76 |
| 4-Ethylphenol | Control | 1 week | 0.030 | * | 1.35 |
| 4-Hydroxy-L-Proline | 3hrs | 2 days | 0.025 | * | -0.66 |
|  | 3hrs | 1 week | 0.020 | * | -0.70 |
| 5-Aminolevulinic Acid | Control | 3hrs | 0.018 | * | 0.52 |
|  | 3hrs | 1 week | 0.044 | * | -0.48 |
| 5-Hydroxyindoleacetate | 3hrs | 2 days | 0.005 | ** | 0.46 |
| 5-Methylcytosine Hydrocloride | 3hrs | 2 days | 0.000 | *** | -0.97 |
|  | 3hrs | 1 week | 0.006 | ** | -0.72 |
| Acetic Acid | Control | 1 week | 0.042 | * | 1.83 |
| Allantoin | 3hrs | 1 week | 0.027 | * | -1.20 |
| Alpha-Hydroxyisobutyric Acid | Control | 2 days | 0.046 | * | 1.33 |
|  | Control | 1 week | 0.004 | ** | 1.40 |
| Azelaic Acid | Control | 1 week | 0.007 | ** | 2.06 |
| Beta-Hydroxyisovaleric Acid | Control | 1 week | 0.023 | * | 1.51 |
| Betaine | Control | 3hrs | 0.032 | * | 0.73 |
|  | 3hrs | 2 days | 0.036 | * | -0.77 |
| C5-Carnitine | Control | 3hrs | 0.019 | * | 0.71 |
|  | 3hrs | 2 days | 0.024 | * | -0.65 |
| D-Fucose | Control | 1 week | 0.038 | * | 1.50 |
| Ethyl-3-Indoleacetate | Control | 3hrs | 0.019 | * | 0.66 |
| Ethylmalonic Acid | Control | 1 week | 0.011 | * | 1.83 |
| Gluconic Acid | 3hrs | 2 days | 0.022 | * | -0.88 |
| Glycogen | Control | 1 week | 0.021 | * | 1.17 |
| Glycolaldehyde Dimer | 3hrs | 1 week | 0.044 | * | -0.23 |
| GTP | Control | 1 week | 0.007 | ** | 1.23 |
| Guanidinoacetic Acid | Control | 1 week | 0.021 | * | 1.16 |
| Hippuric Acid | Control | 1 week | 0.008 | ** | 1.74 |
| Isocitric Acid | Control | 1 week | 0.016 | * | 1.26 |
| L-Alanine | Control | 1 week | 0.014 | * | 1.43 |
| L-Cysteine | Control | 1 week | 0.038 | * | 0.83 |
| L-Glutamic Acid | Control | 1 week | 0.009 | ** | 1.20 |
| L-Glutamine | Control | 1 week | 0.011 | * | 1.05 |
| L-Glutathione-Oxidized | Control | 1 week | 0.007 | ** | 1.11 |
| L-Glycine | Control | 1 week | 0.038 | * | 1.45 |
| L-Isoleucine | Control | 1 week | 0.020 | * | 1.63 |
| L-Lysine | Control | 3hrs | 0.009 | ** | 0.41 |
| L-Methionine | Control | 3hrs | 0.017 | * | -0.39 |
|  | 3hrs | 2 days | 0.013 | * | 0.49 |
| L-Ornithine | Control | 1 week | 0.011 | * | 1.53 |
| L-Phenylalanine | Control | 2 days | 0.046 | * | 0.29 |
| L-Tryptophan | 3hrs | 2 days | 0.011 | * | 0.21 |
|  | 2 days | 1 week | 0.019 | * | -0.18 |
| L-Tyrosine | 3hrs | 2 days | 0.005 | ** | 0.39 |
|  | 3hrs | 1 week | 0.020 | * | 0.36 |
| L-Valine | Control | 1 week | 0.017 | * | 1.48 |
| Levoglucosan | Control | 1 week | 0.031 | * | 1.17 |
| Lysophosphatidylcholine (20-4) | 3hrs | 1 week | 0.023 | * | 0.44 |
| Mannitol | 3hrs | 2 days | 0.016 | * | -0.86 |
| N-Acetyl-L-Aspartic Acid | Control | 1 week | 0.010 | * | 1.15 |
| N-Acetyl-L-Methionine | 3hrs | 2 days | 0.012 | * | 0.54 |
| N-Acetylglycine | Control | 1 week | 0.014 | * | 1.21 |
| N-Methyl-L-Histidine | Control | 3hrs | 0.008 | ** | 0.46 |
| NADP | Control | 1 week | 0.010 | * | 1.24 |
| Trimethyl-L-Lysine | Control | 3hrs | 0.019 | * | 0.28 |
| Nicotinuric Acid | Control | 1 week | 0.003 | ** | 1.73 |
| Phosphocholine (O-12-0-2-0) | 3hrs | 2 days | 0.012 | * | 0.98 |
| Phosphocholine (O-18-4-2-0) | 3hrs | 1 week | 0.016 | * | 0.63 |
| Pyroglutamic Acid | Control | 1 week | 0.038 | * | 1.52 |
| Saccaric Acid | Control | 1 week | 0.010 | * | 1.14 |
| Sebacic Acid | Control | 1 week | 0.011 | * | 1.51 |
| Threonic Acid | Control | 1 week | 0.023 | * | 1.15 |
| TMAO | Control | 1 week | 0.007 | ** | 1.40 |

**Table S2:** metabolites showing significantly different intensities by group-to-group univariate comparisons found in CSF 3hrs, 2 days, and 1 week after acoustically-mediated BBB opening. ** < 0.05, ** < 0.01* and **** 0.001.*

| Metabolites | Groups | | Adjusted p-value | Signif. | Log2(FC) |
| --- | --- | --- | --- | --- | --- |
| 1-Oleoyl-Rac-Glycerol | 2 days | 1 week | 0.026 | * | -0.17 |
| 2-Hydroxypyridine | Control | 3hrs | 0.029 | * | 1.57 |
|  | 3hrs | 1 week | 0.044 | * | -1.43 |
| 3-(4-Hydroxyphenyl)Lactate | Control | 1 week | 0.045 | * | -1.56 |
| 3-Amino-4-Hydroxybenzoic Acid | Control | 3hrs | 0.018 | * | 1.46 |
|  | 3hrs | 2 days | 0.007 | ** | -1.54 |
| 3-Dehydroshikimate | Control | 3hrs | 0.005 | ** | 0.73 |
|  | Control | 1 week | 0.022 | * | 0.58 |
| 3-Hydroxybutanoic Acid | Control | 3hrs | 0.001 | *** | 0.94 |
|  | 3hrs | 1 week | 0.009 | ** | -0.85 |
| 5-Methylcytosine Hydrocloride | Control | 3hrs | 0.000 | *** | 1.29 |
|  | 3hrs | 2 days | 0.013 | * | -1.22 |
| Alpha-Glucose | Control | 1 week | 0.006 | ** | -0.80 |
|  | 3hrs | 1 week | 0.011 | * | -0.75 |
| Citrulline | Control | 3hrs | 0.007 | ** | -2.55 |
| Creatine | Control | 3hrs | 0.012 | * | -0.70 |
| Creatinine | Control | 3hrs | 0.048 | * | 0.38 |
|  | 3hrs | 2 days | 0.026 | * | -0.44 |
|  | 3hrs | 1 week | 0.021 | * | -0.45 |
| Cytidine | 3hrs | 1 week | 0.034 | * | -0.76 |
| Cytosine | Control | 3hrs | 0.039 | * | 0.56 |
|  | 3hrs | 1 week | 0.019 | * | -0.45 |
| Deoxycytidine | Control | 3hrs | 0.025 | * | 0.56 |
|  | 3hrs | 1 week | 0.038 | * | -0.40 |
| Galactitol | Control | 3hrs | 0.002 | ** | 1.08 |
|  | 3hrs | 2 days | 0.028 | * | -0.93 |
|  | 3hrs | 1 week | 0.008 | ** | -0.99 |
| Galactosamine | 3hrs | 1 week | 0.003 | ** | -0.64 |
| Gluconic Acid | 3hrs | 2 days | 0.011 | * | -0.50 |
|  | 3hrs | 1 week | 0.001 | ** | -0.59 |
| Glucuronic Acid | 3hrs | 2 days | 0.007 | ** | -1.46 |
|  | 3hrs | 1 week | 0.026 | * | -1.07 |
| Isocitric Acid | Control | 3hrs | 0.019 | * | 0.91 |
|  | 3hrs | 1 week | 0.031 | * | -0.79 |
| L-Alanine | Control | 3hrs | 0.034 | * | -1.22 |
| L-Allothreonine | Control | 2 days | 0.030 | * | -1.63 |
| L-Arginine | Control | 3hrs | 0.004 | ** | -2.34 |
| L-Asparagine | Control | 3hrs | 0.013 | * | -3.20 |
| L-Glutamine | Control | 3hrs | 0.013 | * | -2.38 |
|  | Control | 2 days | 0.040 | * | -2.01 |
| L-Histidine | Control | 3hrs | 0.029 | * | -0.99 |
|  | 3hrs | 2 days | 0.026 | * | 0.76 |
| L-Serine | Control | 3hrs | 0.022 | * | -1.92 |
| L-Threonine | Control | 2 days | 0.030 | * | -1.63 |
| Lactate | 3hrs | 1 week | 0.017 | * | 0.44 |
| Mannitol | 3hrs | 2 days | 0.002 | ** | -1.03 |
|  | 3hrs | 1 week | 0.003 | ** | -1.03 |
| N-Acetyl-L-Alanine | 3hrs | 1 week | 0.019 | * | -0.45 |
| N-Acetyl-L-Leucine | 3hrs | 1 week | 0.005 | ** | -0.46 |
| N-Acetylglycine | Control | 3hrs | 0.020 | * | 0.35 |
| N-Acetylputrescine | 3hrs | 1 week | 0.021 | * | -0.66 |
| N-Methyltryptamine | Control | 3hrs | 0.004 | ** | -2.36 |
| N-Methyl-L-Histidine | 3hrs | 1 week | 0.006 | ** | -0.85 |
| Trimethyl-L-Lysine | 3hrs | 1 week | 0.006 | ** | -0.31 |
| Nicotinamide | Control | 3hrs | 0.028 | * | 1.53 |
|  | 3hrs | 2 days | 0.028 | * | -1.54 |
|  | 3hrs | 1 week | 0.023 | * | -1.49 |
| Pantothenic Acid | 3hrs | 2 days | 0.004 | ** | 0.59 |
| Rac-Glycerol-1-Myristate | Control | 1 week | 0.020 | * | -0.16 |
|  | 3hrs | 1 week | 0.046 | * | -0.15 |
|  | 2 days | 1 week | 0.046 | * | -0.17 |
| Suberic Acid | Control | 1 week | 0.045 | * | -1.66 |
| Thymidine | Control | 3hrs | 0.008 | ** | 0.53 |
|  | 3hrs | 1 week | 0.019 | * | -0.52 |
| Thymine | Control | 3hrs | 0.007 | ** | 0.93 |
| Uracil | Control | 3hrs | 0.012 | * | 0.42 |
|  | 3hrs | 1 week | 0.004 | ** | -0.49 |
| Uridine | Control | 3hrs | 0.003 | ** | 0.47 |
|  | 3hrs | 2 days | 0.031 | * | -0.37 |
|  | 3hrs | 1 week | 0.005 | ** | -0.45 |
| Xanthine | Control | 3hrs | 0.005 | ** | 0.89 |

**Table S3:** metabolites showing significantly different intensities by group-to-group univariate comparisons found in serum 3hrs, 2 days, and 1 week after acoustically-mediated BBB opening. ** < 0.05, ** < 0.01* and **** 0.001.*

| Metabolites | Groups | | Adjusted p-value | Signif. | Log2(FC) |
| --- | --- | --- | --- | --- | --- |
| 1-Oleoyl-Rac-Glycerol | Control | 1 week | 0.047 | * | -0.29 |
|  | 3hrs | 1 week | 0.013 | * | -0.32 |
| 3-Amino-4-Hydroxybenzoic Acid | 3hrs | 2 days | 0.008 | ** | -1.35 |
| 3-Hydroxybutanoic Acid | Control | 3hrs | 0.008 | ** | 1.45 |
|  | 3hrs | 1 week | 0.000 | *** | -1.74 |
| 3-Methyladipic Acid | Control | 3hrs | 0.007 | ** | 0.74 |
|  | 3hrs | 2 days | 0.036 | * | -0.57 |
|  | 3hrs | 1 week | 0.001 | *** | -1.11 |
| 3-Ureidopropionate | Control | 3hrs | 0.008 | ** | -1.48 |
|  | 3hrs | 2 days | 0.015 | * | 1.35 |
|  | 3hrs | 1 week | 0.018 | * | 1.33 |
| 3.4-Dihydroxy-L-Phenylalanine | 3hrs | 1 week | 0.015 | * | -4.39 |
| 4-Acetamidobutanoate | 3hrs | 2 days | 0.033 | * | -2.20 |
| 4-Coumarate | Control | 3hrs | 0.030 | * | 1.70 |
| 4-Ethylphenol | 3hrs | 1 week | 0.025 | * | -0.33 |
| 4-Methyl-2-Oxo-Pentanoic Acid | Control | 3hrs | 0.002 | ** | 1.71 |
|  | Control | 2 days | 0.041 | * | 1.29 |
|  | 3hrs | 1 week | 0.012 | * | -1.24 |
| 4-Methyl-2-Oxovaleric Acid | Control | 3hrs | 0.005 | ** | 1.75 |
|  | Control | 2 days | 0.050 | * | 1.41 |
|  | 3hrs | 1 week | 0.021 | * | -1.27 |
| 5'-Methylthioadenosine | 3hrs | 2 days | 0.042 | * | -0.56 |
|  | 3hrs | 1 week | 0.004 | ** | -0.68 |
| 5-Hydroxylysine | 3hrs | 1 week | 0.022 | * | 0.55 |
| 5-Oxo-L-Proline | 3hrs | 2 days | 0.011 | * | 1.11 |
|  | 3hrs | 1 week | 0.025 | * | 0.95 |
| Adenine | Control | 3hrs | 0.043 | * | 0.69 |
| Adenosine | 3hrs | 2 days | 0.045 | * | -0.40 |
|  | 3hrs | 1 week | 0.036 | * | -0.42 |
|  | Control | 3hrs | 0.046 | * | -3.29 |
| Alpha-Glucose-1-Phosphate | 3hrs | 2 days | 0.003 | ** | -1.40 |
|  | 3hrs | 1 week | 0.037 | * | -0.88 |
| Betaine | Control | 3hrs | 0.005 | * | -0.43 |
|  | 3hrs | 1 week | 0.023 | *** | 0.56 |
| Bilirubin | 3hrs | 1 week | 0.013 | ** | -1.69 |
| Citramalate | Control | 3hrs | 0.017 | ** | 0.65 |
|  | 3hrs | 1 week | 0.000 | ** | -0.66 |
| Cytidine | 3hrs | 2 days | 0.008 | *** | -0.80 |
|  | 3hrs | 1 week | 0.008 | * | -0.63 |
| Deoxycholate | 3hrs | 1 week | 0.008 | * | -1.58 |
| Ethanolamine Phosphate | 3hrs | 2 days | 0.001 | * | -0.57 |
| Formyl-L-Methionyl Peptide | Control | 3hrs | 0.016 | ** | 0.90 |
|  | 3hrs | 1 week | 0.025 | ** | -0.93 |
| Gluconic Acid | Control | 2 days | 0.047 | ** | -1.07 |
|  | 3hrs | 2 days | 0.002 | * | -0.94 |
| Glucuronic Acid | Control | 2 days | 0.001 | * | -1.41 |
|  | 3hrs | 2 days | 0.006 | **** | -0.29 |
|  | 3hrs | 1 week | 0.017 | ** | -0.32 |
| Glycerate | Control | 3hrs | 0.023 | * | -1.35 |
| Glycine | 3hrs | 1 week | 0.000 | * | 1.45 |
| Glycocholate | 3hrs | 1 week | 0.006 | ** | -1.74 |
| Guanidinoacetate | 3hrs | 2 days | 0.021 | * | 0.74 |
| Indole-3-Acetaldehyde | Control | 1 week | 0.028 | * | -0.57 |
| Inosine | Control | 3hrs | 0.010 | * | -1.11 |
| Isocitric Acid | Control | 3hrs | 0.028 | ** | -1.48 |
|  | 3hrs | 2 days | 0.037 | ** | 1.35 |
|  | 3hrs | 1 week | 0.018 | *** | 1.33 |
| L-Alanine | 3hrs | 1 week | 0.005 | * | -4.39 |
|  | 3hrs | 2 days | 0.005 | * | -2.20 |
| L-Arginine | 3hrs | 2 days | 0.000 | ** | 1.70 |
|  | 3hrs | 1 week | 0.015 | ** | -0.33 |
| L-Asparagine | Control | 3hrs | 0.011 | * | 1.71 |
|  | 3hrs | 2 days | 0.008 | * | 1.29 |
|  | 3hrs | 1 week | 0.006 | * | -1.24 |
| L-Aspartate | 3hrs | 1 week | 0.013 | * | 1.75 |
| L-Carnitine | 3hrs | 1 week | 0.040 | ** | 1.41 |
| L-Glutamic Acid | Control | 3hrs | 0.014 | ** | -1.27 |
|  | 3hrs | 1 week | 0.031 | * | -0.56 |
|  | Control | 3hrs | 0.006 | * | -0.68 |
|  | 3hrs | 2 days | 0.004 | * | 0.55 |
|  | 3hrs | 1 week | 0.015 | * | 1.11 |
| L-Glutamine | Control | 3hrs | 0.016 | * | 0.95 |
|  | 3hrs | 1 week | 0.016 | * | 0.69 |
|  | Control | 3hrs | 0.035 | * | -0.40 |
| L-Isoleucine | 2 days | 1 week | 0.047 | * | -0.42 |
| L-Methionine | 3hrs | 2 days | 0.005 | ** | -0.32 |
| L-Pipecolic Acid | 2 days | 1 week | 0.016 | * | 0.85 |
| L-Proline | 3hrs | 1 week | 0.001 | ** | 0.78 |
|  | 3hrs | 2 days | 0.004 | *** | -0.43 |
|  | 3hrs | 1 week | 0.023 | * | 0.56 |
| L-Serine | 3hrs | 1 week | 0.009 | ** | -1.69 |
|  | 3hrs | 1 week | 0.000 | * | 0.65 |
| L-Threonine | 3hrs | 1 week | 0.022 | * | -0.66 |
| L-Valine | Control | 3hrs | 0.009 | * | -0.80 |
|  | 3hrs | 1 week | 0.011 | *** | -0.63 |
| Lactate | 3hrs | 1 week | 0.035 | * | -1.58 |
| Leucine | 2 days | 1 week | 0.011 | * | -0.57 |
| Linoleate | Control | 3hrs | 0.000 | ** | 0.90 |
|  | 3hrs | 1 week | 0.010 | ** | -0.93 |
| Lyxose | Control | 2 days | 0.022 | * | -1.07 |
|  | 3hrs | 2 days | 0.003 | * | -0.94 |
| Melatonin | Control | 3hrs | 0.009 | ** | -1.41 |
| N-Acetyl-L-Alanine | Control | 3hrs | 0.014 | * | -0.29 |
|  | 3hrs | 1 week | 0.038 | ** | -0.32 |
| N-Acetyl-L-Glutamic Acid | Control | 3hrs | 0.007 | * | -1.35 |
|  | 3hrs | 1 week | 0.015 | * | 1.45 |
| N-Acetyl-L-Leucine | Control | 3hrs | 0.001 | ** | -1.74 |
|  | 3hrs | 1 week | 0.034 | *** | 0.74 |
| N-Acetyl-L-Methionine | Control | 3hrs | 0.024 | * | -0.57 |
| N-Acetyl-L-Phenylalanine | Control | 3hrs | 0.001 | ** | -1.11 |
|  | 3hrs | 1 week | 0.000 | ** | -1.48 |
| N-Acetyl-L-Serine | Control | 3hrs | 0.044 | ** | 1.35 |
|  | 3hrs | 1 week | 0.002 | * | 1.33 |
| N-Acetyl-Tryptophan | 3hrs | 2 days | 0.003 | ** | -4.39 |
|  | 3hrs | 1 week | 0.003 | ** | -2.20 |
| N-Acetylneuraminate | Control | 3hrs | 0.017 | * | 1.70 |
|  | 3hrs | 2 days | 0.007 | ** | -0.33 |
|  | 3hrs | 1 week | 0.006 | ** | 1.71 |
| N-Methyltryptamine | 3hrs | 2 days | 0.049 | ** | 1.29 |
| N-Methyl-L-Histidine | Control | 3hrs | 0.003 | * | -1.24 |
|  | 3hrs | 2 days | 0.005 | ** | 1.75 |
|  | 3hrs | 1 week | 0.003 | ** | 1.41 |
| N-Isopentenyladenine | 3hrs | 1 week | 0.029 | * | -1.27 |
| Trimethyl-L-Lysine | 3hrs | 1 week | 0.004 | * | -0.56 |
| Nicotinamide | Control | 3hrs | 0.007 | ** | -0.68 |
|  | 3hrs | 2 days | 0.025 | *** | 0.55 |
|  | 3hrs | 1 week | 0.017 | ** | 1.11 |
| Palmitate | Control | 3hrs | 0.006 | * | 0.95 |
|  | 3hrs | 1 week | 0.001 | * | 0.69 |
| Phosphocholine | 3hrs | 1 week | 0.004 | ** | -0.40 |
| Putrescine | 3hrs | 2 days | 0.015 | * | -0.42 |
|  | 3hrs | 1 week | 0.014 | *** | -3.29 |
| Pyridoxamine | Control | 3hrs | 0.004 | * | -1.40 |
| Retinoate | Control | 3hrs | 0.028 | ** | -0.88 |
|  | 3hrs | 1 week | 0.001 | ** | -0.32 |
| Sarcosine | 3hrs | 1 week | 0.035 | * | 0.85 |
| Shikimate | Control | 3hrs | 0.002 | * | 0.78 |
|  | 3hrs | 2 days | 0.001 | * | -0.43 |
| Spermidine | Control | 3hrs | 0.037 | * | 0.56 |
|  | 3hrs | 1 week | 0.046 | ** | -1.69 |
| Thymidine | Control | 3hrs | 0.011 | * | 0.65 |
|  | 3hrs | 1 week | 0.034 | * | -0.66 |
| Urate | 3hrs | 2 days | 0.001 | * | -0.80 |
|  | 3hrs | 1 week | 0.038 | * | -0.63 |
| Urocanic Acid | Control | 3hrs | 0.045 | * | -1.58 |

**Table S4:** metabolites showing significantly different intensities by group-to-group univariate comparisons found in urine 3hrs, 2 days, and 1 week after acoustically-mediated BBB opening. ** < 0.05* and *** < 0.01.*

| Metabolites | Groups | | Adjusted p-value | Signif. | Log2(FC) |
| --- | --- | --- | --- | --- | --- |
| 1-Methyl-L-Histidine | Control | 3hrs | 0.043 | * | 3.24 |
|  | 3hrs | 2 days | 0.048 | * | -2.80 |
|  | 3hrs | 1 week | 0.016 | * | -2.83 |
| 2-AminoAdipic Acid | 3hrs | 1 week | 0.018 | * | -0.65 |
| 2-Deoxycytidine | 3hrs | 1 week | 0.005 | ** | -0.61 |
| 2-Oxoglutarate | 3hrs | 1 week | 0.027 | * | 1.59 |
| 2-Picolinic Acid | 3hrs | 1 week | 0.025 | * | -1.09 |
| Allantoin | 3hrs | 1 week | 0.012 | * | -0.25 |
| D-Galactose | 3hrs | 2 days | 0.022 | * | -1.45 |
|  | 3hrs | 1 week | 0.037 | * | -1.47 |
| D-Glucuronic Acid | 3hrs | 1 week | 0.026 | * | -1.07 |
| D-Maltose | Control | 3hrs | 0.046 | * | 1.58 |
|  | 3hrs | 1 week | 0.041 | * | -1.08 |
| Inosine | 3hrs | 2 days | 0.003 | ** | -1.40 |
|  | 3hrs | 1 week | 0.010 | * | -1.15 |
| Isobutyrate | Control | 3hrs | 0.039 | * | 2.28 |
| Kynurenic Acid | 3hrs | 2 days | 0.025 | * | -1.25 |
|  | 3hrs | 1 week | 0.005 | ** | -1.53 |
| L-Arabitol | 3hrs | 1 week | 0.019 | * | -0.83 |
| L-Glutamic Acid | 3hrs | 1 week | 0.049 | * | -0.41 |
| L-Glutamine | 3hrs | 2 days | 0.024 | * | -0.58 |
| L-Leucine | 3hrs | 1 week | 0.019 | * | -0.92 |
| L-Phenylalanine | 3hrs | 1 week | 0.004 | ** | -0.65 |
| L-Proline | 3hrs | 1 week | 0.044 | * | -0.43 |
| Mandelic Acid | 3hrs | 2 days | 0.030 | * | -1.19 |
|  | 3hrs | 1 week | 0.009 | ** | -1.21 |
| Myo-Inositol | 3hrs | 1 week | 0.008 | ** | -1.33 |
| Nicotinuric Acid | 3hrs | 2 days | 0.010 | ** | -1.42 |
|  | 3hrs | 1 week | 0.015 | * | -1.54 |
| Quinolinic Acid | 3hrs | 1 week | 0.011 | * | -0.85 |
| Saccaric Acid | 3hrs | 1 week | 0.005 | ** | -1.03 |
| Taurine | 3hrs | 1 week | 0.025 | * | -1.56 |
| Threitol | Control | 3hrs | 0.003 | ** | 2.27 |
|  | 3hrs | 2 days | 0.031 | * | -1.95 |
|  | 3hrs | 1 week | 0.011 | * | -1.88 |
| trans-Ferulic Acid | 3hrs | 1 week | 0.012 | * | -0.60 |
| UDPG | Control | 3hrs | 0.022 | * | 1.22 |
| Vanillic Acid | 3hrs | 2 days | 0.048 | * | -1.34 |
|  | 3hrs | 1 week | 0.005 | ** | -1.39 |
| Xylitol | 3hrs | 2 days | 0.044 | * | -2.86 |
|  | 3hrs | 1 week | 0.009 | ** | -2.65 |

**Table S5:** Details of intersection between matrices of upset diagram (Figure 2).

| **Intersection** | **Size** | **Metabolites** |
| --- | --- | --- |
| **Striatum – CSF – Serum – Urine** | **60** | L-Glutamic Acid; L-Alanine; Succinate; L-Lysine; L-Aspartate; L-Arginine; L-Glutamine; L-Methionine; L-Ornithine; L-Tryptophane; L-Phenylalanine; L-Tyrosine; Beta-Alanine; Uracil; Glycerol; Fumaric Acid; L-Histidine; Myo-Inositol; L-Proline; L-Asparagine; Glycogen; L-Valine; L-Threonine; D-Glucuronic Acid; Adenosine; Taurine; D-Gluconic Acid; Glyceric Acid; Inosine; Uridine; Isocitric Acid; Spermidine; L-Citrulline; L-Isoleucine; 5-AminoValericAcid; L-Cystine; Trimethylamine; Phosphocholine; Betaine; Creatinine; Saccaric Acid; Pantothenic Acid; 2-Deoxycytidine; Trigonelline; N-Acetyl-L-Aspartic Acid; 3-Hydroxybutyrate; TMAO; 3-Methyl-L-Histidine; L-Anserine; trans-Ferulic Acid; Allantoin; Hippuric Acid; Pyroglutamic Acid; Methylmalonic Acid; Methylguanidine; 3-Hydroxyphenyl Acetic Acid; 3-Phenyl Propionic Acid; alpha-Hydroxyisobutyric Acid; Adipic Acid; Azelaic Acid |
| **Striatum – CSF – Serum** | **64** | Acetic Acid; L-Serine; Adenine; Malate; Nicotinamide; 5'-Methylthioadenosine; Thymine; Thymidine; Guanine; Riboflavin; Lactate; Homoserine; Glycolaldehyde Dimer; Alpha-Glucose; N-Acetylneuraminate; Creatine; L-Carnitine; Glucosamine-6-Phosphate; Urate; Cytosine; Guanosine; Histamine; L-Pipecolic Acid; Trans-Cinnamate; 5,6-Dihydrouracil; Cytidine; Guanidinoacetic Acid; 2-Deoxy-Glucose; Glycerophosphocholine; Oleate; Serotonin; Urocanate; Citramalate; Indole-3-Acetic Acid; D-Fucose; 4-Guanidinobutanoate; 3-(4-Hydroxyphenyl)Pyruvate; Lumichrome; L-Norvaline; 3-Ureidopropionate; N-Acetyl-L-Methionine; N-Acetylputrescine; 10-Hydroxydecanoate; N-Acetyl-L-Phenylalanine; 3-(4-Hydroxyphenyl)Lactate; Tri-methyl-lysine; N6-(Delta2-Isopentenyl)-Adenine; Reichsteins Substance S; Deoxycarnitine; 4-Hydroxy-3-Methoxyphenylglycol; N-Acetylglycine; 5-Hydroxyindoleacetate; 3-Methyl-2-Oxindole; N-Methyltryptamine; Gamma-Linolenic Acid; Diethanolamine; Suberic Acid; Methyl Jasmonate; Nalpha-Acetyl-L-Lysine; Heptanoic Acid; N-Acetyl-L-Serine; Levoglucosan; Beta-Hydroxyisovaleric Acid; 1-Oleoyl-Rac-Glycerol |
| **Striatum – CSF – Urine** | **1** | Sebacic Acid |
| **Striatum – Serum – Urine** | **6** | L-Glycine; GABA; L-Carnosine; 2-AminoAdipicAcid; 2-Propanol; 4-EthylPhenol |
| **CSF - Serum – Urine** | **15** | Pyruvic-Acid; Formate; L-Leucine; 2-Oxoisovalerate; Citrate; D-Maltose; Sarcosine; Glutaric Acid; 2-Deoxyadenosine; trans-4-Hydroxy-L-Proline; Galactitol; Trans-Acotinic Acid; O-Acetyl-L-Carnitine; Homovanillic Acid; Threitol |
| **Striatum – CSF** | **17** | L-Glutathione-oxidized; Hypoxanthine; Xanthine; Mannitol; Tyramine; N-Acetyl-Mannosamine; Pterin; Cortisol; Spermine; Trans-Cinnamaldehyde; 5-Methylcytosine Hydrocloride; Alpha-Tocopherol; 2-Hydroxypyridine; Glucuronolactone; L-Allothreonine; Galacturonic Acid; N-Acetylglycine |
| **Striatum – Serum** | **21** | Amp; Putrescine; Palmitate; 2-Deoxyguanosine; Ethanolamine Phosphate; Levulinic acid; Shikimate; Ll-2,6-Diaminoheptanedioate; Retinoate; 4-Hydroxy-L-Proline; 3-(2-Hydroxyphenyl)Propanoate; 1-Aminocyclopropane-1-Carboxylat; Xanthosine; Cholesteryl Acetate; N-Acetyl-Tryptophan; Glycolic Acid; Dopamine; 3-Methoxy-4-Hydroxymandelate; Glyceraldehyde-3-Phosphate Diethyl acetal; Ethylmalonic Acid; Choline chloride |
| **Striatum – Urine** | **3** | Nicotinic Acid; Threonic Acid; Nicotinuric Acid |
| **CSF – Serum** | **35** | D-Glucose; Choline; D-Mannose; Ethanolamine; Acetone; 4-Methyl-2-Oxo-Pentanoic Acid; L-Kynurenine; 3,4-Dihydroxy-L-Phenylalanine; Thiamine; Malonate; Pyridoxamine; N-Acetyl-L-Glutamic Acid; Epinephrine; Salicylate; 4-Coumarate; D-Glucose-6-Phosphate; Caffeate; Melatonin; Corticosterone; Leukotriene B4; 1-Methyladenosine; N-Acetyl-L-Leucine; Formyl-L-Methionyl Peptide; Malic Acid; N,N-Dimethyl-1,4-Phenylenediamine; N-Acetyl-L-Alanine; Indoxylsulfate; Caprylic Acid; Theophylline; 3-Amino-4-Hydroxybenzoic Acid; Paraxanthine; 2-Methylglutaric Acid; 3-Methyglutaric Acid; Methyl-Indole-3-Acetate; 2-Acetamido-2-Deoxy-Beta-Glucosyl |
| **CSF – Urine** | **4** | Lactose; Dihydrothymine; 1-Methylhydantoin; Dimethylsulfone |
| **Serum – Urine** | **21** | UDPG; L-Glutathione-reduced; 2-Oxobutyrate; Acetoacetate; Methylamine; Xylitol; Hypotaurine; 4-HydroxyphenylAceticAcid; Valerate; D-Galactose; 1,3-Diaminopropane; Dimethylglycine; Kynurenic Acid; Mandelic Acid; 2-Aminobutyric Acid; Isobutyrate; Argininosuccinic Acid; Phenethylamine; Vanillic Acid; Isovaleric Acid; Syringic Acid |


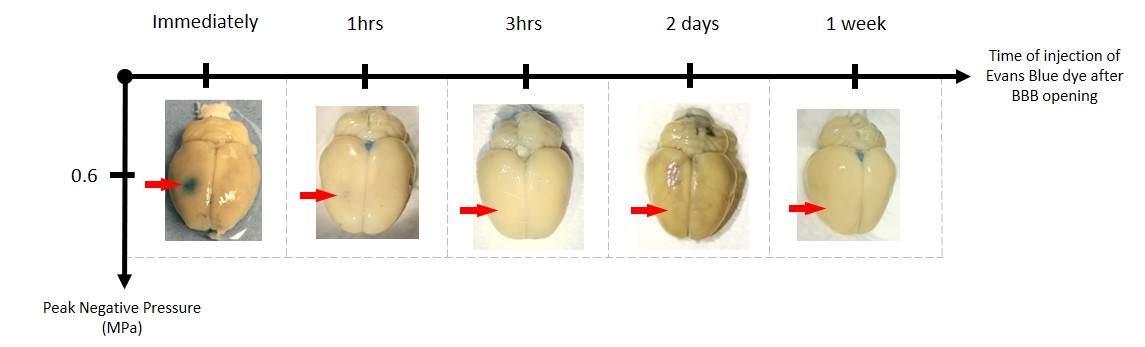


**Figure S1:** Assessment of acoustically-mediated blood-brain barrier opening (BBBO). To evaluate the kinetics of BBB closing, an intravenous bolus of Evans blue (EB; 5mL/kg at 2% w/v) dye was injected immediately, at 1 hrs, 3 hrs, 2 days, and 1 week after the application of microbubble-assisted ultrasound. The rats were intracardiacally perfused with saline and brains were dissected.
